# Mitochondrial priming and response to BH3 mimetics in “one-two punch” senogenic-senolytic strategies

**DOI:** 10.1038/s41420-025-02379-y

**Published:** 2025-03-07

**Authors:** Júlia López, Àngela Llop-Hernández, Sara Verdura, Eila Serrano-Hervás, Eva Martinez-Balibrea, Joaquim Bosch-Barrera, Eduard Teixidor, Eugeni López-Bonet, Begoña Martin-Castillo, Josep Sardanyés, Tomás Alarcón, Ruth Lupu, Elisabet Cuyàs, Javier A. Menendez

**Affiliations:** 1 Program Against Cancer Therapeutic Resistance (ProCURE), Catalan Institute of Oncology, Girona, Spain; 2 Metabolism and Cancer Group, Girona Biomedical Research Institute (IDIBGI), Girona, Spain; 3 Program Against Cancer Therapeutic Resistance (ProCURE), Catalan Institute of Oncology, Badalona, Spain; 4 CARE Program, Germans Trias i Pujol Research Institute (IGTP), Badalona, Spain; 5 Precision Oncology Group (OncoGir-Pro), Girona Biomedical Research Institute (IDIBGI), Girona, Spain; 6 Medical Oncology, Catalan Institute of Oncology, Girona, Spain; 7 Department of Medical Sciences, Medical School, University of Girona, Girona, Spain; 8 Department of Anatomical Pathology, Dr. Josep Trueta Hospital of Girona, Girona, Spain; 9 Unit of Clinical Research, Catalan Institute of Oncology, Girona, Spain; 10 Centre de Recerca Matemàtica (CRM), Barcelona, Spain; 11 Institució Catalana de Recerca i Estudis Avançats (ICREA), Barcelona, Spain; 12 Departament de Matemàtiques, Universitat Autònoma de Barcelona, Barcelona, Spain; 13 Division of Experimental Pathology, Department of Laboratory Medicine and Pathology, Mayo Clinic, Rochester, MN USA; 14 Mayo Clinic Cancer Center, Rochester, MN USA; 15 Department of Biochemistry and Molecular Biology Laboratory, Mayo Clinic Laboratory, Rochester, MN USA; 16 Present Address: Biochemistry and Molecular Biology Unit, Department of Biology, University of Girona, Girona, Spain

**Keywords:** Cancer metabolism, Translational research

## Abstract

A one-two punch sequential regimen of senescence-inducing agents followed by senolytic drugs has emerged as a novel therapeutic strategy in cancer. Unfortunately, cancer cells undergoing therapy-induced senescence (TIS) vary widely in their sensitivity to senotherapeutics, and companion diagnostics to predict the response of TIS cancer cells to a specific senolytic drug are lacking. Here, we hypothesized that the ability of the BH3 profiling assay to functionally measure the mitochondrial priming state—the proximity to the apoptotic threshold—and the dependencies on pro-survival BCL-2 family proteins can be exploited to inform the sensitivity of TIS cancer cells to BH3-mimetics. Replicative, mitotic, oxidative, and genotoxic forms of TIS were induced in *p16*-null/*p53*-proficient, *BAX*-deficient, and *BRCA1*-mutant cancer cells using mechanistically distinct TIS-inducing cancer therapeutics, including palbociclib, alisertib, doxorubicin, bleomycin, and olaparib. When the overall state of mitochondrial priming and competence was determined using activator peptides, the expected increase in overall mitochondrial priming was an exception rather than a generalizable feature across TIS phenotypes. A higher level of overall priming paralleled a higher sensitivity of competent TIS cancer cells to BCL-2/BCL-xL- and BCL-xL-targeted inhibitors when comparing TIS phenotypes among themselves. Unexpectedly, however, TIS cancer cells remained equally or even less overally primed than their proliferative counterparts. When sensitizing peptides were used to map dependencies on anti-apoptotic BCL-2 family proteins, competent TIS cancer cells appeared to share a dependency on BCL-xL. Furthermore, regardless of senescence-inducing therapeutic, stable/transient senescence acquisition, or genetic context, all TIS phenotypes shared a variable but significant senolytic response to the BCL-xL-selective BH3 mimetic A1331852. These findings may help to rethink the traditional assumption of the primed apoptotic landscape of TIS cancer cells. BCL-xL is a conserved anti-apoptotic effector of the TIS BCL2/BH3 interactome that can be exploited to maximize the efficacy of “one-two punch” senogenic-senolytic strategies.

## Introduction

Understanding and exploiting therapy-induced senescence (TIS) for cancer treatment is one of the major challenges in modern oncology [1–4]. TIS is a tumor-suppressive and immunogenic phenomenon induced by certain anticancer drugs that blocks cancer cell proliferation and facilitates anti-tumor immunity, thus contributing to therapeutic outcomes [5–9]. Persistent accumulation of TIS cancer cells can also promote tumorigenesis by stimulating bystander cell proliferation, acquisition of treatment resistance, and metastatic progression through apoptosis escape and dormancy phenomena [10–13]. TIS in normal tissues also causally contribute to treatment-related side effects such as myelosuppression, fatigue, and cardiovascular dysfunction [1, 7, 14–17]. TIS is now at the basis of a rapidly emerging therapeutic strategy known as the “one-two punch”, which combines anticancer therapies with so-called senolytic drugs. The rationale behind “one-two punch” therapies is that a senescence-inducing first punch (e.g., chemotherapy, tumor bed-boosting radiotherapy, molecularly targeted drugs, and/or immunotherapy) may expose novel acquired vulnerabilities in TIS cancer cells. Once selectively targeted (as a second punch), senotherapeutics can promote their selective clearance in the tumor (thereby preventing the development of therapeutic resistance, tumor recurrence, and metastasis) and in normal tissues (thereby alleviating therapy-related side effects) [1–4, 18–22].

The cell fate decision to avoid apoptotic cell death and undergo senescence involves a significant modification of BCL-2 family protein interactions at the mitochondria. Importantly, it has been proposed that the acquisition of the senescent phenotype occurs at the expense of an increased state of cell death *readiness*. This is due to the formation of stable complexes between anti-apoptotic BCL-2 guardians and BH3-only activators/sensitizers that ensure the survival of TIS cancer cells despite increased stress signaling [23–26]. Cell death occurs when the sequestration capacity of anti-apoptotic BCL-2 proteins is exceeded by the levels of BH3-containing pro-apoptotic interactors. Such a reorganization of BCL-2/BH3 protein networks in TIS cells creates a pharmacological vulnerability to the so-called BH3 mimetics (e.g., ABT-263/navitoclax, ABT-199/venetoclax, A-1331852, S63845). These were originally designed to bind anti-apoptotic proteins (i.e., BCL-2, BCL-xL, BCL-w, MCL-1) to increase the apparent stoichiometry of pro-apoptotic BH3-only proteins. Not surprisingly, BH3 mimetics are currently the most widely used class of senolytic drugs [19, 27–29].

We are rapidly learning that sensitivity to senolytic BH3 mimetics is not necessarily an integral part of senescence and some TIS cancer cells—particularly those generated by non-genotoxic drugs—are refractory to the most studied broad-spectrum senolytic, the dual BCL-xL/BCL-2 inhibitor ABT-263/navitoclax [30–36]. Thus, the development of companion diagnostics capable of predicting the senolytic response of TIS cancer cells to a specific BH3 mimetic drug will be required to fully exploit the anticancer potential of BH3 senolytics as part of a one-two punch strategy. Beyond the abundance of anti-apoptotic BCL-2 proteins in TIS cells, the dynamic evolution of the BCL-2/BH3 interactome at the mitochondria may be critical in determining the heterogeneous responses to BH3 senolytics [37–40]. To address this possibility, we hypothesized here that the pretreatment level of the so-called mitochondrial priming in TIS cancer cells, as defined by the BH3 profiling method, could determine the cell death fate in response to BH3 mimetics. BH3 profiling is a functional tool that assesses mitochondrial susceptibility to mitochondrial outer membrane permeabilization (MOMP) in response to synthetic BH3 peptides that promiscuously or selectively interact with pro-survival BCL-2 family proteins [41–45]. Using either activator or sensitizer peptides, the BH3 profiling assay can determine the potential state of overall mitochondrial priming—proximity to the apoptotic threshold—, apoptotic competence, and map specific dependence on one or more proteins of the anti-apoptotic family.

In this study, we used BH3 profiling to elucidate the apoptotic priming and anti-apoptotic dependencies of TIS cancer cells in different therapeutic and genetic contexts. Despite mechanistic heterogeneity among senescence inducers (i.e., palbociclib, alisertib, doxorubicin, bleomycin, and olaparib) and different genetic backgrounds (i.e., *p16*-null/*p53*-proficient, *BAX*-deficient, and *BRCA1*-mutant), mitochondrial priming was not universally increased in TIS cancer cells. Although increased priming correlated with increased sensitivity to BCL-2/BCL-xL inhibition in certain contexts, TIS cancer cells generally exhibited similar or reduced overall priming compared to their proliferative counterparts, revealing a nuanced apoptotic landscape. Dependency mapping with sensitizing peptides implicated BCL-xL as a critical TIS-associated anti-apoptotic determinant, which was validated by the universal senolytic efficacy of the BCL-xL-specific BH3 mimetics A-1331852 across all TIS phenotypes, regardless of senescence-inducing modality or genetic context. These findings refine our understanding of the apoptotic vulnerability of TIS cancer cells and support the development of “one-two punch” approaches that combine senescence induction with BCL-xL-targeted senolytic interventions.

## Results

### *p16-*null/*p53*-proficient A549 TIS cancer cells share a similar senescence-like phenotype

**Fig. 1:**
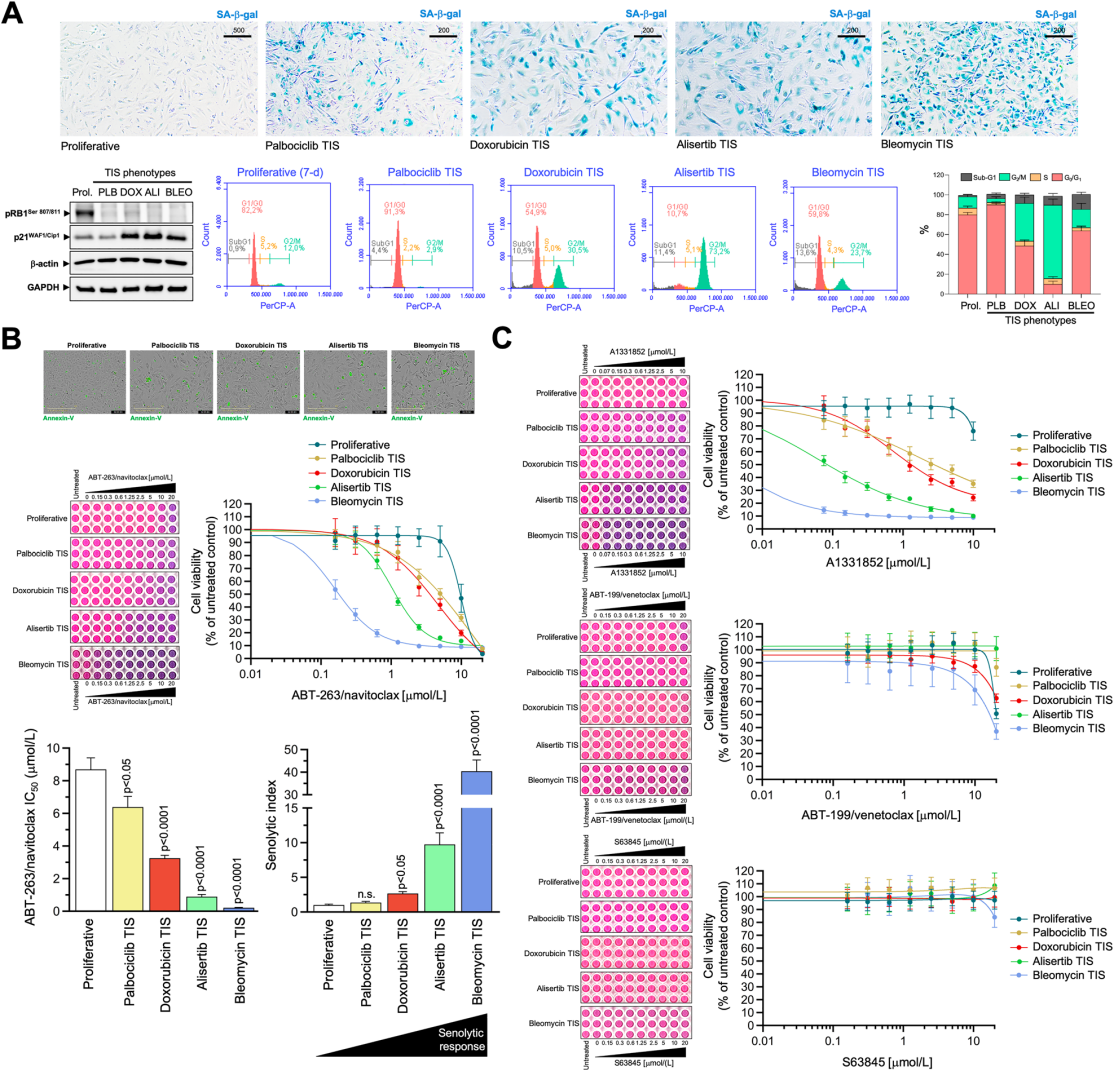
Cell viability responses of A549 TIS lung cancer cells to BH3 mimetics. **A** *Top*. Representative images (*n* = 3) of SA-β-gal staining in A549 lung cancer cells treated with senescence-inducing concentrations of bleomycin, alisertib, doxorubicin or palbociclib for 7 days (scale bar: 500 μm for proliferative/untreated cells; 200 μm for TIS cells). *Bottom left*. Expression levels of phospho-RB^Ser807/Ser811^ and p21^WAF1/Cip1^ were detected by immunoblotting in whole cell lysates of proliferative (untreated) and TIS A549 cancer cells (7-day) using specific antibodies and β-actin/GAPDH as loading controls. The figure shows a representative immunoblot from multiple (*n* = 3) independent experiments (PLB Palbociclib, DOX Doxorubicin, ALI Alisertib, BLEO Bleomycin). *Bottom right*. Representative flow cytometry plots showing the gating of the cell cycle distribution of proliferative (untreated) and TIS phenotypes. The histogram shows the percentage (mean ± S.D., *n* = 3) of cells in the four cell cycle phases as a function of the treatment condition. **B** *Top*. Representative microphotographs of proliferative (untreated) and TIS cancer cells labeled with IncuCyte^®^ Annexin V Green reagent 3 days after harvest and reseeding. *Bottom*. TIS cancer cells and proliferative (untreated) cells were reseeded into 96-well plates (6000 cells/well and 2000 cells/well, respectively) and cultured with increasing concentrations of ABT-263/navitoclax. After 5 days, cell viability was measured using the alarmarBlue™ assay. Shown are representative images of alamarBlue™-based cell viability assays showing changes in the number of metabolically active cells in response to serial dilutions of BH3 mimetics and dose-response curves for ABT-263/navitoclax in proliferative (untreated) and TIS A549 lung cancer cells (untreated = 100%). Bar graphs of the IC_50_ values determined as the μmol/L concentrations of ABT-263/navitoclax required to decrease cell viability by 50%, and the senolytic indexes obtained by dividing the IC_50_ values of ABT-263/navitoclax in proliferative A549 cells by those obtained in TIS A549 cells. Data represent the mean ± S.D. of ≥3 independent experiments performed in triplicate. Statistically significant differences (ANOVA analysis) between proliferative and TIS phenotypes means are shown. n.s. not statistically significant. **C** TIS cancer cells and proliferative (untreated) cells were replated into 96-well plates (6000 cells/well and 2000 cells/well, respectively) and cultured with increasing concentrations of ABT-199/venetoclax, A1331852 or S63845. After 5 days, cell viability was measured using the alarmarBlue™ assay. Shown are representative images of alamarBlue™-based cell viability assays showing changes in the number of metabolically active cells in response to serial dilutions of BH3 mimetics and dose-response curves for ABT-199/venetoclax, A1331852 or S63845 in proliferative (untreated) and TIS A549 lung cancer cells (untreated = 100%). See also Table 1.

The A549 type II-like alveolar lung adenocarcinoma cell line is one of the best and most robust in vitro models to study stress-induced senescence in both cancer and idiopathic pulmonary fibrosis (IPF) [46, 47]. *p16*-null A549 cells, which are known to undergo rapid growth arrest due to the induction of senescence in a p53-dependent manner [48–50], were exposed to four mechanistically distinct and clinically relevant senescence inducers [51], namely: (1) bleomycin, an antibiotic that directly promotes oxidative DNA damage; (2) alisertib (MLN8237), a selective aurora A kinase inhibitor that was identified as one of the most potent pro-senescence agents in a high-throughput compound screen [19, 30, 52]; (3) doxorubicin, a topoisomerase II inhibitor and DNA intercalator [35, 53, 54]; and (4) palbociclib, a selective inhibitor of cyclin-dependent kinases 4 and 6 (CDK4/6), which induces senescence via replication stress and lysosomal trapping [55–57]. Prolonged exposure (7 days) to TIS agents results in a senescence-like phenotype defined by pronounced cytomorphological remodeling (i.e., enlarged, flattened morphology) and increased SA-β-gal activity (up to 80–100% for doxorubicin, alisertib, and bleomycin and up to 60–70% for palbociclib; Fig. 1A, *top*).

A loss of Ser-807/811 retinoblastoma (RB) phosphorylation, a hypophosphorylated and active state of RB that inactivates E2 family transcription factors to arrest cell cycle progression [58–60], was observed on day 7 after treatment with all the TIS agents (Fig. 1A, *bottom*). Except for the palbociclib-TIS phenotype, which exhibited a previously reported downregulation/degradation of the CDK2 inhibitor p21^WAF1/Cip1^ upon chronic palbociclib exposure [61–63], all the other TIS phenotypes were accompanied by a significant accumulation of p21^WAF1/Cip1^ (Fig. 1A, *bottom*). We also confirmed the expected palbociclib-induced G_0_/G_1_ arrest and a significant increase in the number of G_2_/M-arrested cells in response to bleomycin, doxorubicin and especially after treatment with alisertib, further supporting the cellular senescence phenotypes observed by SA-β-gal staining (Fig. 1A, *bottom*). The number of hypodiploid cells in the sub-G_1_ peak ranged from ~5% in the palbociclib-TIS phenotype to ~15% in the bleomycin-TIS phenotype. In addition, staining of reseeded TIS cells in drug-free medium with the Incucyte^®^ Annexin V confirmed the low prevalence of apoptotic events (Fig. 1B, *top*), thereby confirming that the senescence-inducing doses selected to test senolytic responses were largely sublethal.

### *p16-*null/*p53*-proficient A549 TIS cancer cells are resistant to BCL2- and MCL-1-specific inhibitors, but show variable responses to the BCL-X_L_-targeting BH3 mimetics ABT-263/navitoclax and A1331852

We first asked whether phenotypically homogeneous TIS A549 cells were universally sensitive to the widely-used BH3 senolytic ABT-263/navitoclax, a well-established dual BCL-2/BCL-X_L_ inhibitor. We performed bioassays in parental (proliferative) A549 cells and TIS A549 cells using alamarBlue™, a cell viability assay that contains an oxidation-reduction (REDOX) indicator (resazurin) that both fluoresces and changes color in response to the metabolic activity of viable cells. Dose-response assays to graded concentrations of ABT-263/navitoclax showed strong sensitization responses with senolytic indexes (IC_50 proliferative_/IC_50 TIS_) as high as ∼10 and ∼40 in alisertib and bleomycin TIS A549 cells, respectively (Fig. 1B, *bottom*; Table 1). Senolytic indexes in doxorubicin and palbociclib TIS cells were ∼3 and ∼1.5, respectively, indicating a significantly lower sensitization response of these TIS phenotypes to ABT-263/navitoclax compared to proliferative A549 controls (Fig. 1B, *bottom*; Table 1). Therefore, TIS cancer cells with a common senescence-like phenotype show a highly variable response to the dual BCL-2/BCL-X_L_ inhibitor ABT-263/navitoclax.

**Table 1:** Sensitivity of TIS A549 lung cancer cells to BH3 mimetics.

	IC_50_ (μmol/L) ± SD			
	ABT-263/navitoclax	A1331852	ABT-199/venetoclax	S63845
Proliferative (untreated)	8.69 ± 1.23 (1)	>20 (1)	19.28 ± 0.62 (1)	>20 (1)
Palbociclib TIS	6.39 ± 1.14 (1.4)	1.95 ± 1.15 (>10)	>20 (<1)	>20 (1)
Doxorubicin TIS	3.25 ± 0.31 (2.7)	1.31 ± 0.12 (>15)	>20 (<1)	>20 (1)
Alisertib TIS	0.89 ± 0.23 (9.8)	0.06 ± 0.02 (>300)	>20 (<1)	>20 (1)
Bleomycin TIS	0.22 ± 0.03 (39.5)	2.50E-03 ± 0.001 (>8000)	17.07 ± 1.20 (1.1)	>20 (1)

Proliferative and TIS A549 cells were incubated with serial dilutions of BH3 mimetics. The IC_50_ values were determined as the μmol/L concentrations of BH3 mimetics required to decrease cell viability by 50%. Numbers in parentheses are the senolytic indexes (SI) obtained by dividing the IC_50_ values of BH3 mimetics in proliferative A549 cells by those obtained in TIS A549 cells. Data represent the mean ± S.D. of 3 or more experiments.

To further confirm that the heterogeneous responses of TIS cells to BH3 senolytics were not limited to ABT-263/navitoclax, we evaluated the senolytic sensitivity of A549 TIS cells using a broader toolkit of BH3 mimetics, namely the BCL2-specific inhibitor ABT-199/venetoclax, the BCL-X_L_-specific inhibitor A-1331852, and the MCL1-specific inhibitor S63845. AlamarBlue™-based cell viability assays revealed that, although quantitatively different, all TIS phenotypes exhibited a significant senolytic response to the BCL-X_L_-specific inhibitor A1331852. A1331852 IC_50_ values in the low/very low nanomolar range and senolytic indexes as high as >8000 and >300 were observed in alisertib and bleomycin TIS A549 cells, respectively (Fig. 1C, Table 1). A1331852 IC_50_ values in the micromolar range and senolytic indexes tens to hundreds of times lower were observed in doxorubicin and palbociclib TIS A549 cells (Fig. 1C, Table 1). We did not observe any significant senolytic activity of ABT-199/venetoclax and S63845 against either TIS phenotype in A549 cells. Thus, TIS cancer cells generated in response to mechanistically distinct stress inducers share a variable but exquisite response to the BCL-X_L_-specific BH3 mimetic A-1331852.

### Senescent cell behavior contributes to the variability in the sensitivity of *p16-*null/*p53*-proficient A549 TIS cancer cells to BH3 mimetics

**Fig. 2:**
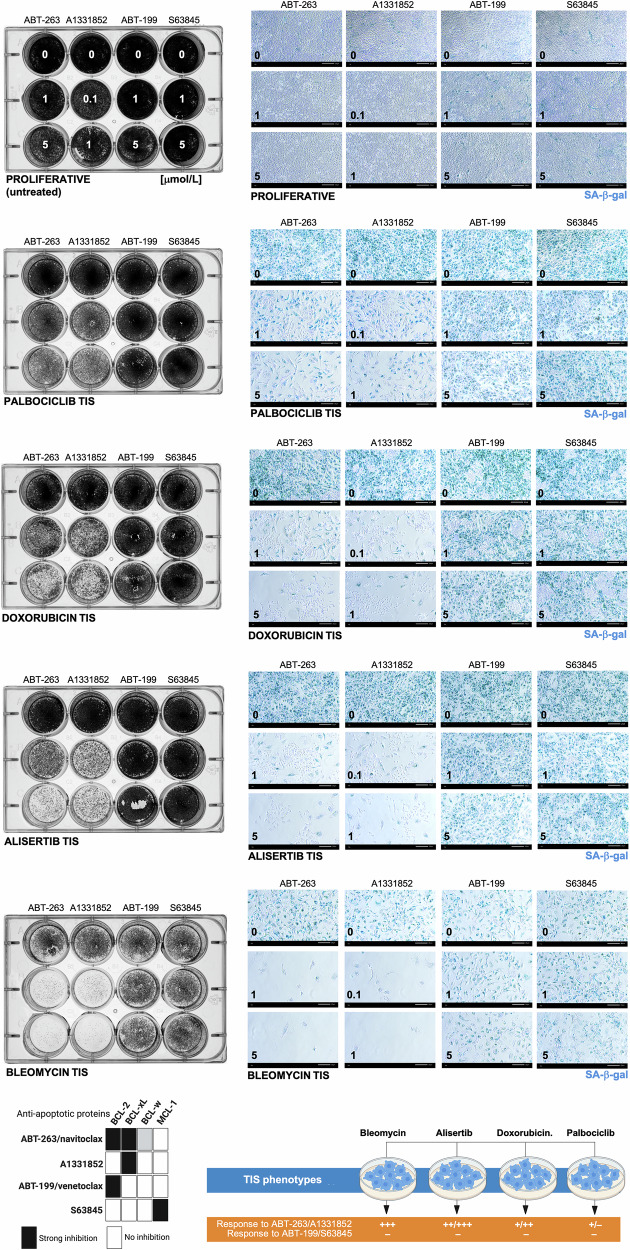
Senolytic responses of A549 TIS cancer cells to BH3 mimetics. A549 lung cancer cells were treated with senescence-inducing concentrations of bleomycin, alisertib, doxorubicin, or palbociclib for 7 days. Senescent cells and proliferative (untreated) cells were replated in 12-well plates (100,000 cells/well and 50,000 cells/well, respectively) and cultured with increasing concentrations of ABT-263/navitoclax, A13318512, ABT-199/venetoclax, or S63845. After 5 days, cell growth and senescence were monitored by crystal violet staining (*left panels*) and SA-β-gal staining (*right panels*). Shown are representative images from three technical replicates. Scale bar = 200 μm. *Bottom left*. BH3 mimetic/anti-apoptotic protein interaction pattern and senolytic sensitivity profile of TIS phenotypes. ABT-263/navitoclax inhibits BCL-2 and BCL-X_L_, A13318512 inhibits BCL-X_L_ only, ABT-199/venetoclax inhibits BCL-2 only, and S63845 inhibits MCL1 only. *Bottom right*. Summary of the responses of A549 TIS cancer cells to BH3 mimetics.

To assess whether cell viability measurements directly correlate with a differential response of senescent versus proliferative cancer cells to BH3 senolytics, we evaluated both the survival and the senescence phenotype of the remaining adherent cells using plaque formation assays and SA-β-gal staining. First, crystal violet staining revealed that the degree of responsiveness of the TIS phenotypes to BH3 mimetics closely paralleled that observed in AlamarBlue™-based cell viability assays (Fig. 2, *left panels*). Thus, all the TIS phenotypes responded exquisitely to the BCL-X_L_-targeting ABT-263/navitoclax and A1331852 BH3 mimetics except for the less sensitive palbociclib-TIS cancer cells. None of the TIS phenotypes responded to the BCL2- and MCL-1-specific inhibitors ABT-199/venetoclax and S63845 in terms of plaque formation. The number of SA-β-gal-positive cells was completely suppressed in all the TIS phenotypes in response to ABT-263/navitoclax and A1331852, whereas a small but observable population of SA-β-gal-positive cells was still present after treatment in palbociclib TIS cells. ABT-199/venetoclax and S63845 failed to significantly decrease the number of SA-β-gal-positive cells in none of the A549 TIS phenotypes (Fig. 2, *right panels*). These results further support the notion that phenotypically indistinguishable A549 TIS cancer cell populations, at least in terms of positive SA-β-gal staining, exhibit a marked variability in their senolytic response to BCL-X_L_-targeting versus BCL2-/MCL-1-specific BH3 mimetics (Fig. 2, *bottom panel*).

### The overall mitochondrial priming in *p16-*null/*p53*-proficient A549 TIS cancer cells is not necessarily higher than in proliferative counterparts

**Fig. 3:**
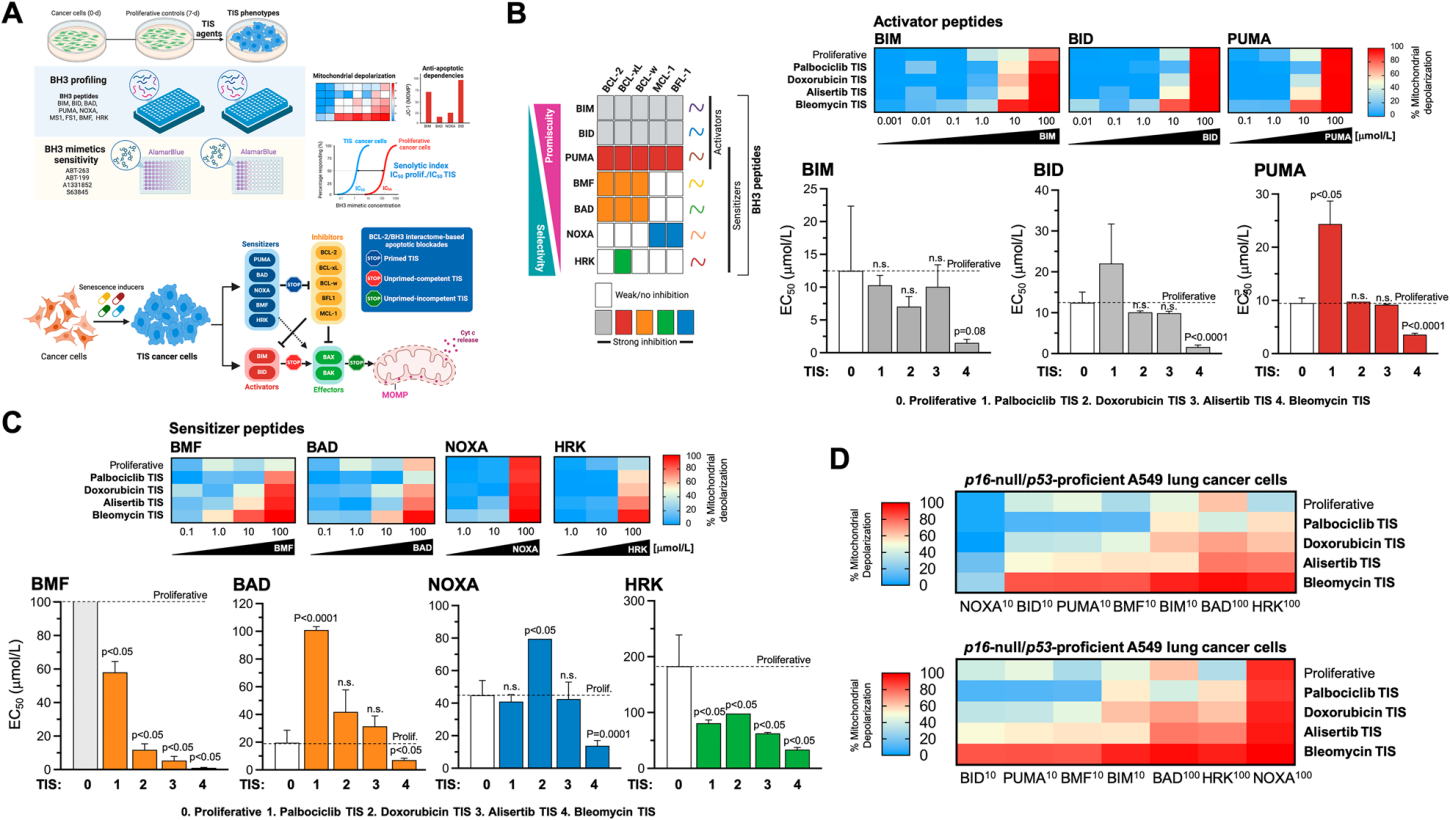
Mitochondrial priming and apoptotic dependencies of A549 TIS lung cancer cells. **A** *Top*. Both the level of mitochondrial priming—the proximity to the mitochondrial apoptotic threshold that determines the ability of TIS mitochondria to initiate apoptosis in response to BH3 peptides—and the nature of apoptotic blockade in TIS cancer cells—the distinct patterns of dependence on pro-survival BCL-2 proteins in the BCL2/BH3 interactome—were assessed using the plate-based JC-1 BH3 profiling. After proliferative (untreated) and TIS cancer cells are permeabilized with digitonin to allow BH3 peptides to diffuse into the cells and interact with intact mitochondria, the loss of JC-1 red fluorescence caused by depolarization of the mitochondrial transmembrane potential (a surrogate for the endpoint MOMP) allows real-time kinetic measurements of the loss of mitochondrial integrity. Primed mitochondria respond more robustly to both activator and sensitizer BH3 peptides and are more susceptible to apoptosis. The pattern of response to BH3 peptides can also identify the functional dynamics between pro- and anti-apoptotic proteins in maintaining cell survival in the TIS phenotypes (e.g., defects in pro-apoptotic signaling or increased addiction to anti-apoptotic proteins). Cell viability analysis using a panel of BH3 mimetics (ABT-263, ABT-199, A13318512, S63845) was used to assess senolytic indexes (SI), which were defined as the ratio of IC_50_ values of proliferative cancer cells to IC_50_ values of TIS cancer cells. *Bottom*. Apoptotic blocks based on BH3 profiling. After exposure to individual activator or sensitizer BH3 peptides, BH3 profiling can distinguish three major blocks (stop signs) through which cells can avoid apoptosis, namely: primed, unprimed-competent, and unprimed-incompetent. *Bottom*. Patterns of interaction between the anti-apoptotic proteins present in cells (*columns*) and the pro-apoptotic synthetic peptides or drugs (*rows*) used in the BH3 profiling assay and the BH3 mimetics cell viability toolkit. BIM, BID, and PUMA inhibit all the inhibitors and are pan-sensitizers. PUMA (shown in red rows) can act as an activator of BAX and BAK. Orange colors highlight those peptides/drugs that inhibit the BCL-2 protein, including BMF and BAD, Blue rows highlight the dependency on mantle cell lymphoma (MCL)-1, whereas NOXA inhibits MCL-1 and BFL-1. Green indicates BCL-X_L_ dependency, as HRK is primarily a BCL-X_L_ inhibitor, but can also inhibit other anti-apoptotic proteins with lower affinity (**A** and **B**, created with Biorender.com*)*. BH3 profiling was performed to measure mitochondrial depolarization in proliferative (untreated) and TIS A549 lung cancer cells exposed to activator (**B**) and sensitizer (**C**) BH3 peptides. Figure shows heat maps of % mitochondrial depolarization caused by increasing concentrations of the activator (BIM, BID, PUMA) and sensitizer (BMF, BAD, NOXA, HRK) peptides in proliferative (untreated) and palbociclib, doxorubicin, alisertib, and bleomycin TIS cells. Data shown are the mean of ≥3 independent experiments using three technical replicates for each peptide. Graphs represent the means (*columns*) ± S.E.M. (*bars*) of ≥3 independent experiments BH3 peptide EC_50s_ (μmol/L) in proliferative and palbociclib, doxorubicin, alisertib, and bleomycin TIS cells. Statistically significant differences (ANOVA analysis) between proliferative and TIS phenotypes means are shown. n.s. not statistically significant. **D** Global heat maps of % mitochondrial depolarization caused by selected concentrations of activator and sensitizer peptides in A549 lung cancer cells. Samples are ordered according to depolarization by the HRK peptide. Data shown are the mean of ≥3 independent experiments with three technical replicates for each peptide.

We hypothesized that the heterogeneous responses of TIS cells to BH3 mimetics may be determined by their relative levels of overall mitochondrial priming—i.e., how close a cell is to the apoptotic threshold determined by MOMP—(Fig. 3A, *top*) and/or by the occurrence of a specific type of apoptotic blockade—i.e., primed and dependent on specific anti-apoptotic proteins, unprimed but competent for apoptosis, or unprimed and incompetent for apoptosis [41–43] (Fig. 3A, *bottom*). We used the so-called BH3 profiling, a functional assay based on short incubations with synthetic BH3 peptides derived from the BH3 domain of the pro-apoptotic BH3-only proteins to induce activation of MOMP (and the consequent depolarization of the mitochondrial transmembrane potential), thereby allowing real-time kinetic measurements of mitochondrial integrity loss [42–45]. The dose required to induce MOMP is inversely correlated with the degree of mitochondrial priming, i.e., the more primed the cells, the more sensitive their mitochondria will be to the synthetic BH3 peptide probes used in the assay [64–68]. Because BH3-only proteins can be either promiscuous or selective in their affinity for anti-apoptotic BCL-2 family proteins, BH3 profiling can also distinguish between promiscuous overall priming and a more selective measure of anti-apoptotic dependence.

BIM or BID peptides can bind to any of the antiapoptotic proteins and can also directly bind and activate BAX and/or BAK. The PUMA BH3 peptide can bind to any of the antiapoptotic proteins but cannot directly activate BAX/BAK (Fig. 3B, *left*). Therefore, the BIM, BID, or PUMA peptides can be used to determine the overall level of mitochondrial priming because the dose required to induce MOMP is inversely correlated with the level of mitochondrial priming (e.g., a high dose of BIM/BID/NOXA required indicates that the cell has a low level of overall priming and *viceversa*). Peptide titration experiments with activator BH3 peptides (BIM, BID, and PUMA) revealed marked differences in the response of TIS phenotypes over a wide range of peptide concentrations. The 50% effective concentration (EC_50_) values for BIM, BID, and PUMA were ~7 to 11 times lower in the most sensitive TIS phenotype, i.e., bleomycin TIS, compared to the less sensitive phenotype, i.e., palbociclib TIS (Fig. 3B, *right*). This means that mitochondria on bleomycin TIS cells have a small reserve of unbound pro-survival proteins and are quickly and completely depolarized by even low doses of activator peptides and are therefore considered to be primed for apoptosis. However, all other TIS phenotypes were similarly or even significantly less primed than their parental (non-senescent, proliferative) counterparts, especially in the case of palbociclib TIS cancer cells. This means that mitochondria on palbociclib TIS cells have a large reserve of unbound pro-survival proteins and only respond to high doses of activator peptides. Thus, the expected rule of a higher level of overall priming underlying the acquisition of sensitivity of TIS cancer cells to (BCL-xL-targeting) BH3 senolytics appears to hold when comparing TIS phenotypes among themselves, but does not hold when considering the mitochondrial priming status of proliferative controls.

### *p16-*null/*p53*-proficient A549 TIS cancer cells are dependent on BCL-xL for survival

Using the BH3-only sensitizer peptides, which can selectively bind specific anti-apoptotic proteins to release the sequestered activator peptides but can also directly activate BAX/BAK in the absence of potent direct activators (BIM/BID) to cause MOMP, the BH3 profiling assay can detect if a cell has a particular dependence on one or more pro-survival BCL-2 family proteins to actively block apoptosis. Peptide titration experiments with the BH3-only sensitizer peptides BAD, NOXA, HRK, and BMF revealed that the extent of depolarization differed significantly between the different TIS phenotypes (Fig. 3C, *top*). The EC_50_ values for BAD, NOXA, HRK, and BMF were ~3–55 times lower in the most sensitive (bleomycin) TIS phenotype than in the less sensitive (palbociclib) TIS phenotype (Fig. 3C, *bottom*), suggesting that ABT-263/A1331852-sensitive TIS cells are highly dependent on a robust anti-apoptotic signaling compared to ABT-263/A1331852-resistant TIS cells. More importantly, when compared to proliferative counterparts, all the TIS phenotypes showed lower EC_50_ values for the sensitizer peptides BMF, which binds potently to BCL-2 and BCL-xL but can also bind and directly activate BAK, and HRK, which is mainly a selective inhibitor of BCL-xL. When the EC_50_ values of each (activator and sensitizer) BH3 peptide determined in the BH3 profiling assay were compared with the IC_50_ values of ABT-263/navitoclax and A1331852 against proliferative control cells and A549 TIS phenotypes, we observed a perfect positive correlation with BMF (*r*^2^ = 1; *p* = 0.0167; Tables S1, S2) and good correlations with HRK (*r*^2^ = 0.9; *p* = 0.083; Tables S1, S2). When the EC_50_ values of each BH3 peptide were compared with the senolytic indexes of ABT-263/navitoclax and A1331852, we also found a significant positive correlation not only with BMF but also with BIM (Tables S3, S4). A global heat map representation of % mitochondrial depolarization in A549 TIS cells clearly highlighted that distinct apoptotic priming and blockade profiles appear to distinguish the senolytic response to ABT-263/navitoclax and A1331852 among the TIS phenotypes themselves (Fig. 3D). When considering the proliferative counterparts, our results suggest that the acquisition of a primed apoptotic landscape associated with an exquisite senolytic response of *p16-*null/*p53*-proficient A549 TIS cancer cells to ABT-263/navitoclax and A1331852 is the exception rather than the rule.

### *BAX*-incompetent LoVo TIS cancer cells exhibit senolytic responses to the BCL-xL-specific inhibitor A1331852

Inactivation or downregulation of the pore-forming proteins BAX and/or BAK prevents the occurrence of MOMP, and cells using this apoptotic escape mechanism would be identified when none of the BH3 peptides (activators or sensitizers) induce MOMP and are termed “apoptotic refractory” or “incompetent” for intrinsic apoptosis [43, 68]. A delineation of the transcriptome and senolytic responses in a broad panel of 13 cancer cell lines induced to TIS by alisertib and etoposide—the so-called cancer SENESCopedia—recently revealed that the most unresponsive model to ABT-263/navitoclax was the colon cancer cell line LoVo [30], which harbors biallelic frameshift mutations in *BAX* [69, 70]. Therefore, we took advantage of *BAX*-deficient LoVo cells to test the hypothesis that an unprimed, incompetent state of TIS cancer cells may inform pan-refractoriness to BH3 senolytics.

**Fig. 4:**
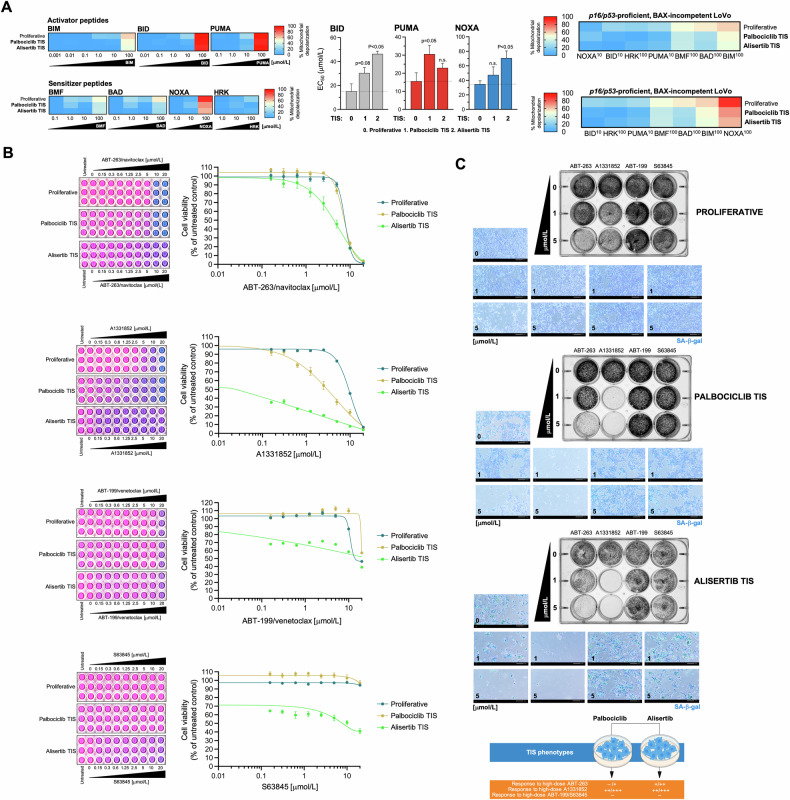
Mitochondrial priming and response to BH3 mimetics in BAX-incompetent TIS models of LoVo colon cancer cells. **A** *Left*. BH3 profiling was performed to measure mitochondrial depolarization in proliferative (untreated) and TIS LoVo colon cancer cells exposed to activator and sensitizer BH3 peptides. Figure shows heat maps of % mitochondrial depolarization caused by increasing concentrations of the activator (BIM, BID, PUMA) and sensitizer (BMF, BAD, NOXA, HRK) peptides in proliferative (untreated) and palbociclib and bleomycin TIS cells. Data shown are the mean of ≥3 independent experiments using three technical replicates for each peptide. Graphs represent the means (*columns*) ± S.E.M. (*bars*) of ≥3 independent experiments BH3 peptide EC_50s_ (μmol/L) in proliferative (untreated) and palbociclib and alisertib TIS cells. Statistically significant differences (ANOVA analysis) between proliferative and TIS phenotypes means are shown. n.s. not statistically significant. *Right*. Figure shows global heat maps of % mitochondrial depolarization caused by selected concentrations of activator and sensitizer peptides in LoVo colon cancer cells. Samples are ordered according to depolarization by the BIM peptide. Data shown are the mean of ≥3 independent experiments with three technical replicates for each peptide. **B** TIS cancer cells and proliferative (untreated) cells were replated into 96-well plates (7,000 cells/well and 4,000 cells/well, respectively) and cultured with increasing concentrations of ABT-263/navitoclax, A1331852, ABT-199/venetoclax, or S63845. After 5 days, cell viability was measured using the alarmarBlue™ assay. Shown are representative images of alamarBlue™-based cell viability assays showing changes in the number of metabolically active cells in response to serial dilutions of BH3 mimetics and dose-response curves for ABT-263/navitoclax, A1331852, ABT-199/venetoclax, or S63845 in proliferative (untreated) and TIS LoVo cancer cells (untreated=100%). See also Table 2. **C** *Top*. LoVo colon cancer cells were treated with senescence-inducing concentrations of alisertib or palbociclib for 7 days. Senescent cells and parental (proliferative) cells were replated into 12-well plates (200,000 cells/well and 150,000 cells/well, respectively) and cultured with increasing concentrations of ABT-263/navitoclax, A1331852, ABT-199/venetoclax, or S63845. After 5 days, cell growth and senescence were monitored using crystal violet staining (*left panels*) and SA-β-gal staining (*right panels*). Shown are representative images from three technical replicates. Scale bar = 200 μm. *Bottom*. Summary of the responses of LoVo TIS cancer cells to BH3 mimetics.

*p16/p53*-proficient but *BAX*-deficient LoVo cells were exposed to the senescence inducers alisertib and palbociclib (Fig. S1), and the apoptotic priming of the corresponding TIS phenotypes was measured using the BH3 profiling method. With the exception of the highest concentrations of the BID, PUMA, and NOXA peptides, we observed that MOMP-related mitochondrial depolarization was generally low in *BAX*-deficient LoVo cells, regardless of the proliferative or TIS state (Fig. 4A). Indeed, an unprimed state was even more pronounced in the palbociclib and alisertib TIS phenotypes of *BAX*-deficient LoVo cells when compared to proliferative counterparts (Fig. 4A).

We then tested the senolytic response of palbociclib and alisertib TIS LoVo cells to ABT-263/navitoclax, ABT-199/venetoclax, A1331852, and S63845. AlamarBlue™-based cell viability assays confirmed that the “unprimed-incompetent” TIS phenotype of LoVo cells exhibited a marked resistance to the senolytic effects of ABT-263/navitoclax, ABT-199/venetoclax and S63845 (Fig. 4B). However, it was noteworthy that they acquired a senolytic response to the BCL-X_L_-specific inhibitor A1331852 (Fig. 4B). Indeed, a senolytic index as high as ~900 was observed when comparing the low nanomolar concentrations of A1331852 required to kill alisertib-induced senescent LoVo cells with the micromolar concentrations required to kill proliferative LoVo cells; a less pronounced but significant 3-fold increase in responsiveness to A1331852 was also observed in palbociclib TIS LoVo cells (Table 2). Plaque formation and SA-β-gal staining for all of the BH3 mimetics confirmed that the (sensitive versus resistant) behavior of senescent cells, and not that of SA-β-gal-negative non-senescent proliferative cells, indeed contributed to the exquisite senolytic response of TIS LoVo cells to high but therapeutically relevant concentrations of A1331852 (1 μmol/L; Fig. 4C). Therefore, an unprimed, incompetent state does not accurately reflect a pan-refractory response to BH3 senolytics in *BAX*-deficient TIS cancer cells.

**Table 2:** Sensitivity of TIS LoVo colon carcinoma cells to BH3 mimetics.

	IC_50_ (μmol/L) ± SD			
	ABT-263/navitoclax	A1331852	ABT-199/venetoclax	S63845
Proliferative (untreated)	7.56 ± 1.19 (1)	8.98 ± 1.61 (1)	19.37 (1)	>20 (1)
Palbociclib TIS	7.35 ± 1.45 (1)	2.82 ± 0.28 (3.2)	>20 (<1)	>20 (1)
Alisertib TIS	4.63 ± 1.61 (1.6)	0.01 ± 0.01 (898)	13.89 (1.4)	>20 (1)

Proliferative and TIS LoVo cells were incubated with serial dilutions of BH3 mimetics. The IC_50_ values were determined as the μmol/L concentrations of BH3 mimetics required to decrease cell viability by 50%. Numbers in parentheses are the senolytic indexes (SI) obtained by dividing the IC_50_ values of BH3 mimetics in proliferative LoVo cells by those obtained in TIS LoVo cells. Data represent the mean ± S.D. of 3 or more experiments.

Overall, BH3 functional profiling of mechanistically heterogeneous TIS phenotypes in A549 (*p16*-null and *p53/BAX*-proficient) and LoVo (*p16/p53*-proficient and *BAX*-deficient) cancer cells revealed the occurrence of the three major BCL-2/BH3 interactome categories, namely: primed, unprimed-competent, and unprimed-incompetent. While increased mitochondrial priming was associated with greater sensitivity to BCL-xL-targeting BH3 mimetics in certain contexts, this increase was the exception rather than a general feature across TIS phenotypes. Importantly, the use of a chemical inhibitor toolkit revealed a consistent senolytic response to the BCL-xL-specific BH3 mimetic A1331852, even in TIS cancer cells—both competent and incompetent—lacking enhanced mitochondrial priming.

### The unprimed olaparib-induced TIS phenotype in *BRCA1*-deficient cancer cells is responsive to the BCL-xL-specific inhibitor A1331852

Entry into TIS is not necessarily a permanent endpoint, but rather a state of variable intensity and depth that may be transient in nature [71, 72]. Therefore, we finally sought to determine whether the apparent pan-sensitivity of TIS cancer cells to BCL-xL-specific BH3 mimetics regardless the mitochondrial priming status can also occur when the acquisition of the TIS phenotype is transient. We chose a therapeutically relevant scenario in which DNA damage due to pharmacological inhibition of poly(ADP-ribose) polymerase 1 (PARP1), an enzyme essential for detecting DNA breaks and initiating various forms of DNA repair, induces a transient senescent-like phenotype that is restricted to the context of homologous recombination (HR) deficiencies, such as those caused by *BRCA1/2* mutations [73–75].

**Fig. 5:**
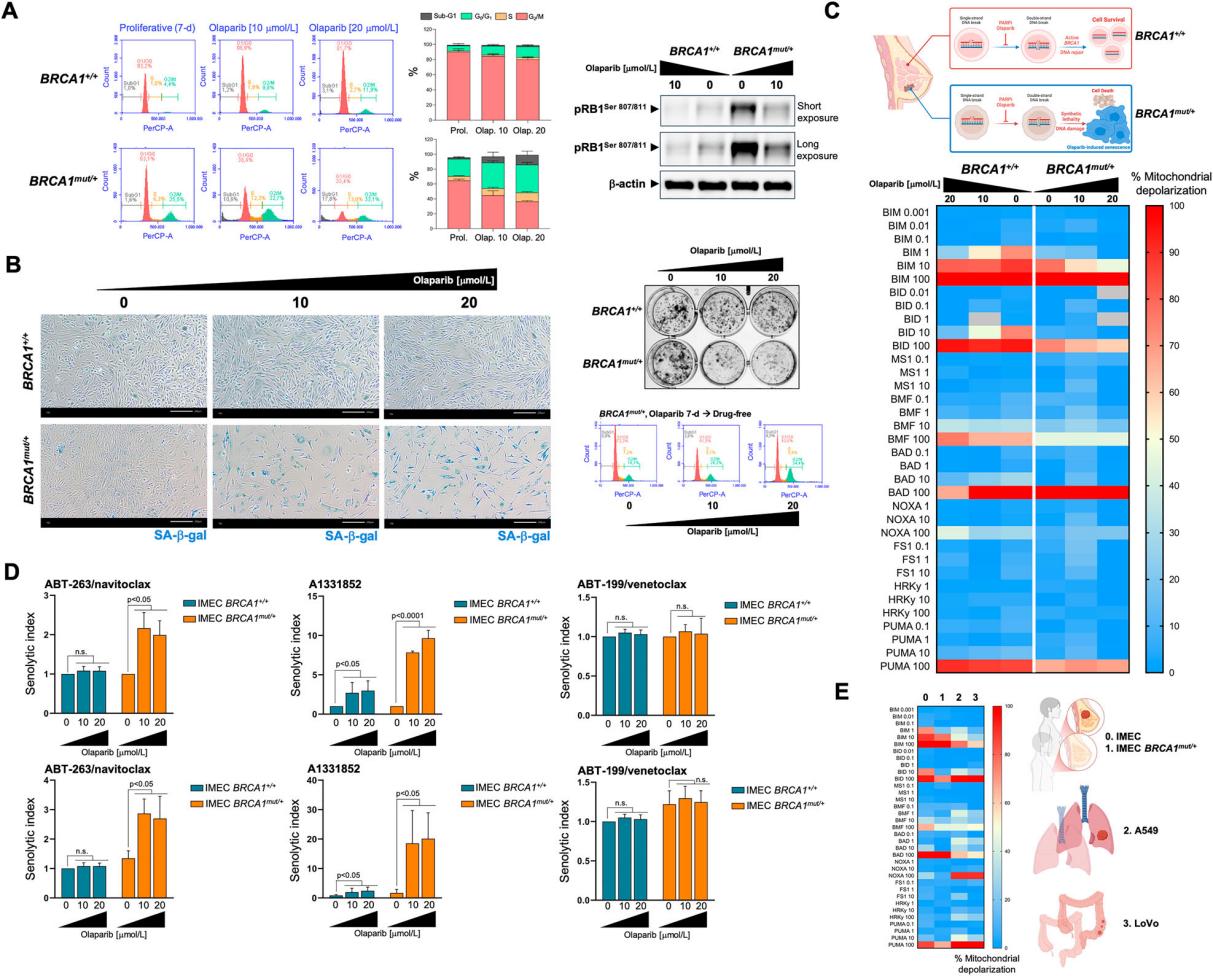
Mitochondrial priming and response to BH3 mimetics in the transient olaparib-induced TIS phenotype in *BRCA1*-mutant breast epithelial cells. **A** *Left*. Representative flow cytometry plots showing the gating of the cell cycle distribution of proliferative (untreated) and olaparib-treated IMEC *BRCA1*^*+/+*^ and IMEC *BRCA1*^*mut/+*^ cells. The histograms show the percentage (mean ± S.D., *n* = 3) of cells in the four cell cycle phases as a function of the treatment condition. *Right*. Expression levels of phospho-RB^Ser807/Ser811^ and p21^WAF1/Cip1^ were detected by immunoblotting in whole cell lysates of proliferative (untreated) and olaparib-treated IMEC *BRCA1*^*+/+*^ and IMEC *BRCA1*^*mut/+*^ cells (7-day) using specific antibodies and β-actin/GAPDH as loading controls. The figure shows a representative immunoblot from multiple (n = 3) independent experiments. **B**. *Left*. Representative images of SA-β-gal staining in proliferative (untreated) and olaparib-treated IMEC *BRCA1*^*+/+*^ and IMEC *BRCA1*^*mut/+*^ cells (7-day) from three independent experiments (scale bar: 200 μm). *Right top*. Recovery of proliferative potential was evaluated by reseeding untreated and olaparib-treated IMEC *BRCA1*^*+/+*^ and IMEC *BRCA1*^*mut/+*^ cells (7-day) cultures (2000 cells/well) in drug-free conditions. After 10 days, cell growth was monitored by crystal violet staining. Shown are representative images from three technical replicates. *Right bottom*. Representative flow cytometry plots showing the gating of the cell cycle distribution olaparib-treated IMEC *BRCA1*^*mut/+*^ cells reseeded in drug-free conditions for 3 days. **C** *Top*. The synthetic lethal interaction between the PARPi olaparib and DNA repair due to *BRCA1* deficiency triggers breast epithelial cancer cell senescence. *Bottom*. BH3 profiling was performed to measure mitochondrial depolarization in proliferative (untreated) and olaparib-treated IMEC *BRCA1*^*+/+*^ and IMEC *BRCA1*^*mut/+*^ breast epithelial cells exposed to activator and sensitizer BH3 peptides. Figure shows a global heat map of % mitochondrial depolarization caused by increasing concentrations of the activator (BIM, BID, PUMA) and sensitizer (BMF, BAD, NOXA, HRK) peptides in proliferative (untreated) and olaparib-treated cells. Data shown are the mean of ≥3 independent experiments using three technical replicates for each peptide. **D** Proliferative and olaparib-treated IMEC *BRCA1*^*+/+*^ and IMEC *BRCA1*^*mut/+*^ cells (7-day) were incubated with serial dilutions of ABT-263/navitoclax, A1331852, and ABT-199/venetoclax. After 5 days, cell viability was measured using the alarmarBlue™ assay. *Top*. Graphs show the senolytic indexes obtained by dividing the IC_50_ values of BH3 mimetics in proliferative IMEC (*BRCA1*^*+/+*^ and *BRCA1*^*mut/+*^) cells by those obtained in corresponding olaparib-treated IMEC (*BRCA1*^*+/+*^ and *BRCA1*^*mut/+*^) cells. *Bottom*. Graphs show the senolytic indexes obtained by dividing the IC_50_ values of BH3 mimetics in proliferative IMEC (*BRCA1*^*+/+*^ and *BRCA1*^*mut/+*^) cells by those obtained in olaparib-treated IMEC *BRCA1*^*+/+*^ cells. Data represent the mean (*columns*) ± S.D. (*bars*) of 3 or more independent experiments. Statistically significant differences (ANOVA analysis) between the means for untreated and olaparib-treated cells are shown. n.s. not statistically significant. **E** Global heat map of % mitochondrial depolarization in proliferative (untreated) IMEC *BRCA1*^*+/+*^ (normal-like breast epithelium, 0), IMEC *BRCA1*^*mut/+*^ (breast cancer-prone breast epithelium, 1), A549 (lung adenocarcinoma, 2), and LoVo (colon cancer cells, 3).

We took advantage of a *p53*-proficient, non-cancerous breast epithelial cell line (IMEC) immortalized by *hTERT* introduction (*hTERT*-IMEC), in which the pathogenic *BRCA1* mutation *185delAG*—which confers impaired homology-mediated DNA repair and hypersensitivity to genotoxic stress—was heterozygously introduced by gene targeting [76, 77]. *hTERT*-IMEC *BRCA1*^*185delAG/+*^ cells treated for 7 days with the FDA-approved PARP inhibitor olaparib exhibited a G_2_/M arrest accompanied by a significant decrease in the G_0_/G_1_ subpopulation, the appearance of a significant number of hypodiploid cells in the sub-G_1_ peak, and a significant decrease of phospho-RB levels (Fig. 5A). All these features, which were completely absent in *hTERT*-IMEC *BRCA1*^*+/+*^ parental counterparts treated with equimolar concentrations of olaparib, suggested that olaparib-induced DNA damage led to activation of the G_2_-M checkpoint and resulted in cell cycle arrest exclusively in *BRCA1*-mutant cells. Close visual observation suggested that although some *BRCA1*^*185delAG/+*^ cells undergo apoptotic cell death, a significant population of *BRCA1*-mutant cells appeared to undergo a cytostatic response with marked senescence-like cytomorphologic remodeling. Staining for increased β-galactosidase activity confirmed a concentration-dependent increase in SA-β-gal-positive *BRCA1*^*185delAG/+*^ cells at day 7 after olaparib treatment, which largely spared *BRCA1*^*+/+*^ parental counterparts (Fig. 5B, *left*). When untreated and olaparib-treated cells (7 days) were harvested and reseeded at low density (1000–2000 cells/well) under drug-free conditions for 10 days to allow colony formation, we observed that a partial but significant proliferative recovery occurred after olaparib washout in olaparib-TIS *BRCA1*^*185delAG/+*^ cells (Fig. 5B, *right*), confirming the transient nature of the TIS phenotype induced by olaparib [74].

Measurement of overall mitochondrial apoptotic priming based on the response to BIM, BID, and PUMA peptides, revealed that *BRCA1*^*185delAG/+*^ cells required higher concentrations of pro-apoptotic BH3-only activators than *BRCA1*^*+/+*^ parental counterparts to induce MOMP (Fig. 5C). This unprimed state was even more pronounced in olaparib-TIS *BRCA1*^*185delAG/+*^ cells, which showed only apoptotic priming when using the highest concentration of BIM. No dependence on any pro-survival BCL-2 family protein was observed when olaparib-TIS *BRCA1*^*185delAG/+*^ cells were diagnosed with BH3-only sensitizer peptides (Fig. 5C). An AlamarBlue™-based chemosensitivity assessment of senolytic responses to BH3 mimetics revealed a complete refractoriness of olaparib-TIS *BRCA1*^*185delAG/+*^ cells to BCL2-targeted ABT-199/venetoclax and MCL-1-targeted S63845 (*data not shown*) BH3 mimetics but a significant acquisition of senolytic response to the BCL-2/BCL-xL-targeted ABT-263/navitoclax (~2 to 3-fold; Fig. 5D). This acquisition of a senolytic response was even more pronounced when using the BCL-xL-targeting A1331852, as olaparib-TIS *BRCA1*^*185delAG/+*^ cells were up to 10-fold more sensitive than proliferative *BRCA1*^*185delAG/+*^ cells (Fig. 5D, *top*) and up to 20-fold more sensitive than parental *BRCA1*^*+/+*^ cells (Fig. 5D, *bottom*). Interestingly, olaparib-treated *BRCA1*^*+/+*^ parental cells acquired a significantly enhanced responsiveness (up to 3-fold) to A1331852. The acquisition of an exquisite response to the BCL-xL-specific inhibitor A1331852 occurs in the olaparib-induced TIS phenotype regardless of its unprimed state and transient nature.

## Discussion

A two-step strategy combining senescence-inducing anticancer agents with senolytics has emerged as a promising approach to improve the outcomes and mitigate the adverse side-effects of cancer treatment [1–4, 18, 20]. While the rewiring of the BCL-2 interactome in senescent cells is thought to prime them for apoptosis, the universal use of pro-apoptotic BH3 mimetics as the senolytics of choice in “one-two punch” regimens remains uncertain due to the inconsistent responses observed in TIS cancer cells [20–24]. Here, we show that the expected mitochondrial-primed apoptotic state is the exception rather than the rule in a mechanistically diverse set of TIS phenotypes. In contrast to the current paradigm, BH3 profiling reveals that the majority of TIS cancer cells are either unprimed or have reduced mitochondrial priming compared to their proliferative counterparts. However, all TIS phenotypes, regardless of their genetic background or the senescence inducer, show a significant, albeit highly variable, sensitivity to the BCL-xL-selective BH3 mimetic A-1331852. These findings redefine the apoptotic susceptibility of TIS cancer cells and confirm BCL-xL as a critical target to optimize the efficacy of “one-two punch” senogenic-senolytic therapies.

Reliable prediction of senolytic efficacy of BH3 mimetics against TIS cancer cells is still lacking. A robust DNA damage response (DDR) has been associated with sensitivity to the dual BCL-2/BCL-xL inhibitor ABT-263/navitoclax. TIS cancer cells induced by irradiation or genotoxic agents are highly sensitive to ABT-263/navitoclax, whereas those induced by non-genotoxic agents, such as enzalutamide, are refractory despite their senescent phenotype [35]. However, this relationship is not consistent. Palbociclib-induced TIS cancer cells, although known to induce DDR and impair DNA repair [78–80], show significantly lower sensitivity to ABT-263/navitoclax than TIS cells driven by other DNA damaging agents such as alisertib or doxorubicin [81–93]. Furthermore, cancer cells harboring biallelic mutations in the apoptotic effector *BAX* are completely refractory to ABT-263/navitoclax, independent of DDR induction by TIS agents [30]. Even in a *BAX/BAK*-competent scenario, cancer cells and senescent melanocytes that upregulate the expression of anti-apoptotic BCL-2 family members can also remain completely resistant to the senolytic effects of ABT-263/navitoclax and ABT-737/venetoclax [34]. Collectively, these inconsistencies suggest that mitochondrial responsiveness to BH3 mimetics may be determined by the evolution of the BCL-2/BH3 interactome, rather than the nature or intensity of the DDR itself [23]. However, gene expression profiling of the pro- and anti-apoptotic members of the BCL-2 family has not been able to reliably predict the senolytic response of TIS cancer cells [28, 84]. Alternatively, it has been proposed that a functional assessment of the BCL-2/BH3 interactome may be a more accurate approach to predict the selectivity and efficacy of BH3 mimetics as senolytics [42–45, 85–87]. Here, with the goal of providing a refined framework for the rational exploitation of TIS vulnerabilities, we have explored such a proposed utility of BH3 profiling as a functional tool to guide the tailored use of BH3 mimetics in senogenic-senolytic strategies.

The differential responses of replicative, mitotic, oxidative, and genotoxic forms of TIS to BH3 mimetics highlight that sensitivity to senolytics cannot be inferred solely from the presence of a senescence-like cytomorphological phenotype or canonical markers such as SA-β-gal positivity. While A549 TIS lung cancer cells induced by bleomycin and alisertib exhibited exquisite sensitivity to BCL-xL-targeting agents, palbociclib- and doxorubicin-TIS A549 cancer cells were relatively resistant, correlating with their reduced overall priming. Although this finding reinforced the critical role of mitochondrial apoptotic readiness in dictating senolytic efficacy when comparing TIS phenotypes among themselves, contrary to expectations, overall mitochondrial priming status—as determined by BH3 profiling—was not consistently elevated relative to their proliferative counterparts. As expected for cancer cells lacking the functional activity of pro-apoptotic (BAX and/or BAK) effectors, BH3 profiling failed to detect an increased overall mitochondrial apoptotic priming in incompetent TIS phenotypes generated in *BAX*-deficient LoVo colon cancer cells. An unprimed state was also found in the transiently and specifically acquired TIS phenotypes of *BRCA1*-deficient breast epithelial cells exposed to the PARP inhibitor olaparib. Thus, in terms of the potential states of apoptotic priming and competence as determined by BH3 profiling, most TIS phenotypes mimic most therapy-resistant cancer cells that select for reduced mitochondrial priming following either conventional chemotherapy or treatment with pro-apoptotic BH3 mimetics including ABT-263/navitoclax [66]. Instead, a nuanced interplay of senescence-inducing mechanisms and anti-apoptotic dependencies appeared to underlie the variability in susceptibility to the pro-apoptotic effects of BH3 mimetics observed across TIS phenotypes.

Our results implicate BCL-xL, but not BCL-2 or MCL-1, as a central pro-survival factor of TIS cancer cells, as demonstrated by the BH3 profiling-based apoptotic dependency mapping of A549 TIS cancer cells and the nearly universal sensitivity of all the TIS phenotypes tested to the BCL-xL-specific inhibitor A1331852. The discovery that *BAX*-incompetent, unprimed TIS phenotypes in LoVo colon cancer cells retain sensitivity to the BCL-xL-specific BH3 mimetic A1331852 extends our current paradigm of senescence-associated vulnerabilities based on BH3 profiling. This finding demonstrates that loss of *BAX* does not necessarily confer pan-TIS resistance to BH3 mimetics, but further supports the argument that the “activator” activity of the remaining BAK—rather than an intrinsic property of certain BH3-only proteins—may be largely dependent on the pro-survival profile induced by the TIS stressor [88–91]. Similarly, olaparib-induced TIS in *BRCA1*-deficient cancer cells—a model of transient, unprimed senescence—shows remarkable sensitivity to A1331852, further highlighting the versatility of BCL-xL-targeted senolytics against mechanistically heterogeneous TIS states. This finding confirms and extends a previous observation that BCL-xL is the major BCL2 family member underlying the apoptotic resistance of the olaparib-induced senescence-like phenotype in ovarian and triple-negative breast cancer (TNBC) cells [74]. Collectively, these observations challenge the notion that BH3 senolytic efficacy requires a primed, apoptotic-competent mitochondrial state and highlight the pivotal, transversal role of BCL-xL in preserving mitochondrial integrity of TIS cancer cells [92, 93], independent of the senescence-inducing therapeutic, stable or transient acquisition of senescence, or the underlying genetic context.

**Fig. 6:**
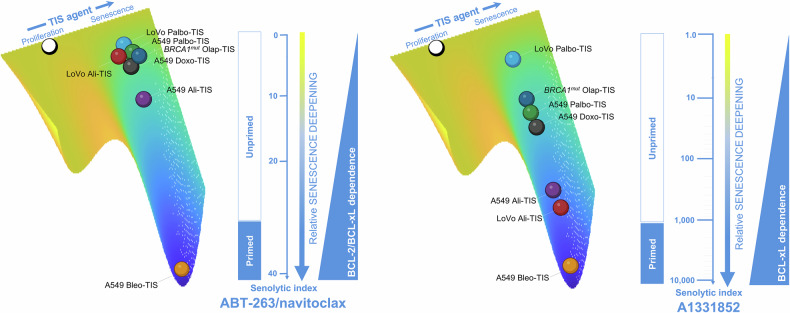
TIS and BCL-2/BCL-xL dependencies revealed by ABT-263/navitoclax and A1331852: Linear and logarithmic scaling of senescence deepening. The figure shows the relative senescence deepening of TIS phenotypes as a function of either combined BCL-2/BCL-xL or selective BCL-xL dependency using ABT-263/navitoclax or A1331852 senolytic indexes as surrogates. The scale for senescence deepening in the context of ABT-263/navitoclax is linear, highlighting incremental differences between genetically diverse cancer cell lines treated with different therapeutic senescence-inducing (TIS) agents. The logarithmic scale in the A1331852 context highlights the exponential differences in BCL-xL-driven TIS modulation, demonstrating a sharper uncoupling of primed versus unprimed mitochondrial states compared to the linear relationship in BCL-2/BCL-xL-driven TIS modulation. The observed uncoupling between primed/unprimed mitochondrial status and BCL-2/BCL-xL dependency (left panel) versus selective BCL-xL dependency (right panel) suggests a hierarchical shift under the selective pressure of TIS agents. A plausible hypothesis is that unprimed cells predominantly rely predominantly BCL-xL as a survival factor, whereas primed cells show a broader dependence on BCL-2 family proteins.

Our findings are consistent with previous evidence implicating BCL-xL as a/the key anti-apoptotic safeguard during oncogene-induced and therapeutic stress-induced senescence [92–95]. Selective pharmacological inhibition of BCL-xL appears to convert or redirect protective senescence-initiating responses to apoptotic cell death in both highly primed and minimally primed TIS cancer cells. Therefore, it may be tempting to suggest that the true determinant of senolytic response is the ability of BH3 mimetics to increase the likelihood of cell death by limiting protective senescence-initiating processes activated by mitochondrial apoptotic stress [92]. We propose that TIS phenotypes can be viewed as a continuum toward a full-fledged arrest state of variable strength and depth in mitochondrial priming, BCL-xL-dependency, and responsiveness to navitoclax/A1331852 (Fig. 6). In those TIS phenotypes where senescence-underlying maintenance mechanisms can permit cell cycle re-entry if not continuously provided (e.g., olaparib, palbociclib), BCL-xL dependency appears to be decoupled from an overt mitochondrial priming. These phenotypes are poorly responsive to the dual BCL-2/BCL-xL inhibitor ABT-263/navitoclax, but show a significant response to the BCL-xL-specific BH3 mimetic A1331852. In those TIS phenotypes that are in or near to the deep attractor of a stable senescence arrest state (e.g., alisertib, bleomycin), a strict BCL-xL dependence is coupled with an increased mitochondrial priming. These phenotypes are good responders to the dual BCL-2/BCL-xL inhibitor ABT-263/navitoclax and gain an exquisite synthetic lethal response to A1331852. Thus, ∼40- and >2500-fold differences in the senolytic index values of ABT-263/navitoclax and A1331852, respectively, can be observed across the continuum of strength and senescence deepening of mechanistically heterogeneous TIS phenotypes (Fig. 6). Future studies should evaluate how selective knockout of BCL-2 and/or BCL-xL would affect senescence deepening, cytochrome c release, and survival in primed and unprimed states. Whether the underlying molecular propensity and/or ability to translate the analog/graded strength and depth of the senescent phenotype induced by therapeutic perturbations into a digital on/off cell fate decision between unprimed and primed states (and thus from poor to highly responsive to BCL-xL-targeting senolytics) is related to the baseline mitochondrial priming of the cell/tissue-of-origin (Fig. 5E) is an intriguing possibility worth exploring in future studies.

From a therapeutic perspective, our study advances the feasibility of BCL-xL-specific drugs as a cornerstone of “one-two punch” senogenic-senolytic strategies. The exceptional potency of A1331852 against multiple TIS models, independent of apoptotic priming or competence, highlights its robustness as a potential senolytic agent compared to ABT-263/navitoclax. Although selective targeting of BCL-xL may help to avoid the broader toxicities associated with dual BCL-2/BCL-xL inhibitors and provide a more precise therapeutic window to eliminate TIS cancer cells while sparing normal tissues, it could be argued that the BCL-xL-related platelet effect of A1331852 may complicate its clinical use. However, as observed here in combination with alisertib or bleomycin, or in other studies in combination with the multi-TKI regorafenib [78], A1331852 may be effective as a senolytic agent at very low concentrations, most likely before thrombocytopenia can become limiting. Indeed, the undesirable platelet effects of BCL-xL-targeting BH3 mimetics can be mitigated by careful dosing. Based on the discovery that the combination of the PARPi olaparib and the senolytic ABT-263/navitoclax is an effective one-two punch strategy in a mouse model of ovarian cancer [74], the Exactis-03 Phase I clinical trial is designed to evaluate the safety and the recommended dose of combined treatment with olaparib + ABT-263/navitoclax in women with TNBC who have a somatic or germline mutation in *BRCA1/2* and in women with recurrent high-grade serous ovarian cancer (HGSC) who have progressed on platinum-containing chemotherapy, with intermittent administration of ABT-263/navitoclax to prevent its hematologic toxicity [79]. In this study, olaparib be administered alone for 14 days and then continuously for 28 days at a fixed dose, while the dose of navitoclax will be escalated. This Phase I study will define the recommended Phase II dose (RP2D) for a future Phase II study that will formally evaluate the one-two punch of olaparib (senogenic)-navitoclax (senolytic) in TNBC and HGSC patients (NCT05358639). Our findings in a *BRCA1*-deficient, non-tumorigenic basal-like/TNBC breast epithelial model confirm and further suggest that senescence-targeting combination therapies with A1331852 may represent promising, more effective alternatives for the clinical implementation of a BCL-xL-targeted one-two punch approach in patients with specific genomic alterations in mechanisms of homology-directed DNA repair. The recent design of a BCL-xL proteolysis-targeting chimera (PROTAC), which targets BCL-xL to the Von Hippel-Lindau (VHL) E3 ligase for degradation, would allow for safer and more potent senolytic activity, but expected lower platelet toxicity than either ABT-263/navitoclax or A1331852, as VHL is poorly expressed in platelets [80, 96–99]. During the preparation of the final version of this manuscript, TIS melanoma cells were found to share a BCL-xL-mediated pro-survival adaptation underlying the exquisite senolytic activity of A1331852 and the PROTAC BCL-xL degrader DT2216 [93]. Collectively, these data highlight the importance of BCL-xL as a/the maintenance molecular determinant of TIS cancer cells and the potential to circumvent the side effects associated with BCL-xL-targeted therapies in translating one-two punch senogenic-senolytic strategies from experimental models to the clinical arena.

We acknowledge several concerns and weaknesses with our current approach. First, the *on-target* mechanism of action and therapeutically relevant concentration ranges of all the BH3 mimetics used in this study are critical to the robustness of our findings. The BH3 mimetics ABT-263/navitoclax, A1331852, ABT-199/venetoclax, and S63845 were previously found to possess mitochondrial-dependent killing only by competitive binding to their reported targets at the mitochondria using a panel of cell lines encompassing the most common anti-apoptotic dependencies and apoptosis-resistant BAX/BAK double knockouts, and confirmed by the so-called mito-priming method to engineer BCL2 dependency [100, 101]. Importantly, the concentration ranges of BH3 mimetics used included 1 μmol/L, which is approximately the serum concentration achieved by the FDA-approved ABT-199/venetoclax when used as recommended [102]. Although the most potent anti-TIS inhibitor A1331852 has shown some off-target cell killing effects [100], these occurred at concentrations as high as 10 μmol/L, which were not accounted for in our experimental approach. Second, our assessment of mitochondrial determinants of senolytic responses among TIS phenotypes exclusively applies to BH3 mimetics, recently classified as class I senolytics [23]. However, class II senolytics that inhibit survival pathways such as p53 and AKT may also affect BH3 network reorganization. *TP53* mutations affect the sensitivity of TIS cancer cells to BH3 mimetics, as p53 transcriptionally regulates BH3-only proteins and interacts with BCL-2 family proteins at the MOM [29, 103–105]. Similarly, AKT signaling promotes senescent cell survival by inhibiting the pro-apoptotic protein BAD and stabilizing the anti-apoptotic MCL-1 [106]. Notably, class III senolytics, which disrupt dysregulated cellular homeostasis in senescent cells, may indirectly engage apoptotic pathways, as seen with cardiac glycosides such as ouabain and digoxin [107, 108] which induce the pro-apoptotic NOXA. Future studies should test whether the tested role of mitochondrial (un)priming and/or BCL-xL dependence is not limited to BH3 mimetics but also applies to other classes of senolytics. Third, and more importantly, the timing of exposure to senolytics relative to the induction of senescence and the dynamic shifts in the BH3 protein network from early to late senescence have been shown to significantly influence sensitivity to senolytics [109, 110]. Therefore, a 7 days period from exposure to senogenic therapeutics to BH3 functional profiling likely enables the occurrence of complex pro-survival adaptations in the BH3:BCL-2 apoptotic interactome. This might complicate the actual relevance of BH3 profiling as a pharmacodynamic biomarker of the senolytic response of TIS cancer cells to BH3 mimetics. Future studies using dynamic BH3 profiling (DBP) after short-term exposure of TIS cancer cells to BH3 mimetics [86] or microscopy-based, single-cell resolution methods such as high-throughput DBP [111] would help to identify drug susceptibilities and the occurrence of intra-TIS heterogeneity priming phenomena in senolytic-resistant TIS phenotypes.

## Conclusion

Using the BH3 profiling assay, our study systematically dissects the mitochondrial apoptotic landscape of TIS cancer cells to challenge the prevailing assumption that BH3 mimetics universally exploit increased mitochondrial priming in senescent cells. Contrary to expectations, our findings underscore the limitations of mitochondrial priming as a pan-predictive marker of sensitivity to BH3 senolytics and redefine the apoptotic vulnerability of TIS cancer cells. The near-universal efficacy of BCL-xL-specific senolytics such as A1331852 in diverse senescent contexts highlights a conserved therapeutic vulnerability that can be exploited to enhance and optimize the efficacy of “one-two punch” strategies. Future efforts should focus on elucidating how and why BCL-xL dependency may be shared across other TIS phenotypes and the development of companion diagnostics to identify patients who are most likely to benefit from BCL-xL-targeted senolytic therapies.

## Materials and methods

### Cell lines and culture

The human cell lines A549 (ATCC CCL-185), LoVo (ATCC CCL-229), and HEK293T (ATCC CRL-3216) were obtained from the ATCC (Manassas, VA, USA). Cells were routinely expanded in Dulbecco’s modified Eagle’s medium (DMEM, Gibco) supplemented with 10% heat-inactivated fetal bovine serum (FBS; Linus), 1% L-glutamine, 1% sodium pyruvate, 50 IU/mL penicillin, and 50 μg/mL streptomycin. Human *BRCA1* (185delAG/+) MCF10A cells with heterozygous knock-in of a 2-bp deletion of *BRCA1* resulting in a premature termination codon at position 39 and MCF10A isogenic parental cells were obtained from Horizon Discovery Ltd., Cambridge, UK (Cat# HD 101–018 and HD PAR-058, respectively). Cells were routinely grown in DMEM/F-12 (Gibco, Life Technologies, Paisley, UK) including 2.5 mmol/L L-glutamine, and 15 mmol/L HEPES, supplemented with 5% horse serum (HS), 10 μg/ mL insulin, 20 ng/mL hEGF, 0.5 μg/mL hydrocortisone and 0.1 μg/ mL cholera toxin.

Cells were grown at 37 °C in a humidified atmosphere containing 5% CO_2_ and were in the logarithmic growth phase at the start of the experiments. Cell lines were authenticated by STR profiling, both performed by the manufacturer and confirmed in-house at the time of purchase according to ATCC guidelines. Cells were passaged by starting a low-passage cell stock every month until to 2–3 months after resuscitation. Cell lines were screened for mycoplasma contamination using a PCR-based method for *Mycoplasma* detection prior to experimentation and were intermittently tested thereafter.

### Drugs and reagents

ABT-263/navitoclax (Cat. #S1001), ABT-199/venetoclax (Cat. #S8048), A1331852 (Cat. #S7801), S63845 (Cat. #S8383), doxorubicin (Cat. #S1208), and PD-0332991/palbociclib (Cat. #S1116) were purchased from Selleckchem (Houston, TX, USA). MLN8237/alisertib (Cat. #331-10890-1) and bleomycin sulfate (Cat. #331-11727-3) were purchased from RayBiotech Inc. (Norcross, GA, USA). AlamarBlue™ Cell Viability Reagent (Cat. #DAL1100), JC-1 dye (mitochondrial membrane potential probe; Cat. #T3168), PageRuler™ Plus Prestained Protein Ladder (Cat. #26619), FITC Annexin V/Death Cell Apoptosis Kit with FITC annexin V and PI for flow cytometry (Cat. #V13242) and 7-AAD (7-aminoactinomicin D; Cat. #A1310) were purchased from Thermo Fisher Scientific Inc. (Waltham, MA, USA). Antibody against p21^WAF1/CIP1^ (12D1; Cat. #2947) was purchased from Cell Signaling Technology, Inc. (Danvers, MA, USA). Antibodies against phospho-RB1 (Ser807/811; Cat.# 30376-1-AP), β-actin (#66009-1-Ig) and GAPDH (#60004-1-Ig) were purchased from Proteintech Group, Inc. (Rosemont, IL, USA). Incucyte^®^ Annexin V Dye for apoptosis (Cat. #4642) was purchased from Sartorius (Göttingen, Germany). BH3 peptides with HPLC purity >95% were custom-made and purchased from Genecust EUROPE (France) in 2- or 5-mg scale. The senescence β-galactosidase staining kit (Cat. #9860) was purchased from Cell Signaling Technology (Danvers, MA, USA).

### Therapy-induced senescence

We identified optimal initial cell densities and senescence-inducing concentrations as those that allowed A549, LoVo, and MCF10A *BRCA1*^*+/+*^/MCF10A *BRCA1*^*mut/+*^ cell cultures to reach ~70–80% confluence after 7 days of treatment, while promoting the appearance of the major classical markers of senescence, namely enlarged and flattened cell shape and increased senescence-associated β-galactosidase (SA-β-gal) activity in the highest number of cells. Treatment with optimal senescence-inducing concentrations of bleomycin (20 μmol/L), alisertib (500 nmol/L), and doxorubicin (50 nmol/L) resulted in 80–100% SA-β-gal-positive population. Treatment with palbociclib (5 μmol/L) resulted in 60–70% SA-β-gal-positive populations. Treatment of MCF10A *BRCA1*^*+/+*^/MCF10A *BRCA1*^*mut/+*^ cell lines with olaparib (10 and 20 μmol/L) resulted in 60–70% SA-β-gal-positive populations specifically in *BRCA1*^*mut/+*^ cells. Cellular β-galactosidase activity was detected using the Senescence β-Galactosidase Staining Kit (Cell Signaling Technology, Danvers, MA, USA) according to the manufacturer’s instructions.

### Immunoblotting

Cells were washed with ice-cold phosphate-buffered saline, and scraped immediately after the addition of 100 µL of 2% SDS, 1% glycerol, and 5 mmol/L Tris-HCl, pH 6.8 supplemented with protease and phosphatase inhibitors. Protein lysates were collected in 1.5 mL microcentrifuge tubes and samples were sonicated for 1 min (in an ice bath) with 2 s sonication at 2 s intervals to completely lyse cells and reduce viscosity. Protein content was determined using the Bradford protein assay (Bio-Rad, Hercules, CA, USA). Sample buffer was added and extracts were boiled at 100 °C for 5 min. Equal amounts of protein were electrophoresed on 15% SDS-PAGE gels, transferred to nitrocellulose membranes, and incubated with primary antibodies as indicated, followed by incubation with a horseradish peroxidase-conjugated secondary antibody and chemiluminescence detection. GADPH and β-actin were used as protein loading controls. Chemiluminescence detection images were acquired using a ChemiDocMP imaging system (Bio-Rad). Uncropped original images were merged with the corresponding protein size ladder.

### Cell cycle and apoptosis detection

Cell cycle distribution using 7-ADD as analyzed on an Accuri C6 flow cytometer, and data were analyzed using Accuri C6Flow software. Apoptotic cells were labeled with Incucyte^®^ Annexin V green dye (1:200 dilution) and imaged using “phase” and “green” image channels in the Incucyte S3 Cell-by-Cell Analysis System.

### Senolytic index

Cell viability was measured using alamarBlue™ assays. The non-toxic, cell-permeable alamarBlue™ (resazurin) reagent is an oxidized form of a redox indicator that is blue in color and non-fluorescent. Upon entering living cells, the reagent is reduced to resorufin, which changes color from blue to red and becomes fluorescent. The oxidized environment upon loss of cell viability maintains the non-fluorescent, blue color of resazurin.

To calculate senolytic indexes, TIS A549 and LoVo cancer cells and their proliferative counterparts were harvested after 7 days of treatment with TIS inducers, reseeded in 96-well plates (2000 cells/100 μL/well proliferative A549 cells vs 6000 cells/100 μL/well TIS A549 cells; 4000 cells/100 μL/well proliferative LoVo cells vs 7000 cells/100 μL/well TIS LoVo cells; 2000 cells/100 μL/well in both proliferative and olaparib TIS MCF10A *BRCA1*^*+/+*^/MCF10A *BRCA1*^*mut/-*)^ and exposed to graded concentrations of BH3 senolytics for an additional 5 days. Cells were incubated with the alamarBlue solution (10 μL/well) at 37 °C for 4 h and the increase in fluorescence signal was measured using a fluorescence detector. The senolytic index was defined as the ratio between the inhibitory concentration 50 (IC_50_) values of proliferative cells and the IC_50_ values of bleomycin-, alisertib-, doxorubicin-, palbociclib-, and olaparib-induced TIS cancer cells. The IC_50_ values were defined as the concentration of drug that produced a 50% reduction in control fluorescence (by interpolation).

### BH3 profiling

The JC-1 plate-based BH3 profiling was performed according to the original procedure provided by the Letai lab at the Dane-Farber Cancer Institute (https://letailab.dana-farber.org/bh3-profiling.html). Briefly, BIM, BID, BMF, PUMA, BAD, HRK, NOXA, and PUMA peptides (0.01/0.1–100 μmol/L), alamethicin (25 μmol/L), and CCCP (10 μmol/L) were added to JC-1 MEB staining solution (150 mmol/L mannitol, 10 mmol/L HEPES-KOH, 50 mmol/L KCl, 0.02 mmol/L EGTA, 0.02 mmol/L EDTA, 0.1% BSA, 5 mmol/L succinate, pH 7.5) in a black 384-well plate. Single cell suspensions from proliferative and 7-day TIS cell cultures were prepared in JC-1-MEB buffer including 0.0025% digitonin and kept at RT for 10 min to allow for cell permeabilization and dye equilibration. After adding 2–3 × 10^4^ cells/well to the 384-well plate, fluorescence will be measured at 590 nm emission/545 nm excitation using a microplate reader at 30 °C every 15 min for a total of 3 h to obtain kinetic traces for each peptide. Percent depolarization was calculated by normalization to the AUC of the solvent-only control (DMSO, % depolarization) to generate the final profiles.

### Statistical analysis

All cell-based observations were confirmed by at least three independent experiments performed in triplicate for each cell line and for each condition. Data are expressed as mean ± SEM or ±SD. Bar graphs, curves, and statistical analyses were generated using GraphPad Prism 10 (GraphPad Software, San Diego, CA). Comparisons of means of ≥3 groups were performed by ANOVA, and the existence of individual differences, in the case of significant *F* values in ANOVA, was tested by Dunnett’s multiple contrasts. *p* < 0.05 were considered to be statistically significant. All statistical tests were two-tailed.

## Supplementary information

Supplemental Information

Original data

## Data Availability

All of the data sets used in the present study are available from the corresponding authors upon reasonable request.
