# Supplementary material for: Mitochondrial priming and response to BH3 mimetics in “one-two punch” senogenic-senolytic strategies
Source: Cell Death Discov. 2025 Mar 7;11:91. doi: 10.1038/s41420-025-02379-y (PMC11889205; doi:10.1038/s41420-025-02379-y)
Supplement: Supplementary file 1 — Supplemental Information [file 41420_2025_2379_MOESM1_ESM.docx]

**Mitochondrial priming and response to BH3 mimetics in**

**“one-two punch” senogenic-senolytic strategies**

**–SUPPLEMENTARY MATERIAL–**

**1. SUPPLEMENTARY TABLES**

**Table S1. Correlation between mitochondrial priming (EC_50_ values for BH3 peptides-induced MOMP) and senolytic sensitivity (ABT-263/navitoclax IC_50_) in proliferative (untreated) TIS A549 lung cancer cells.**

|  | **BIM** | **BID** | **PUMA** | **BAD** | **NOXA** | **HRK** | **BMF** |
| --- | --- | --- | --- | --- | --- | --- | --- |
| **Spearman r** | 0.9 | 0.9 | 0.4 | 0.4 | 0.5 | 0.9 | 1.0 |
| **P value** | 0.0833 | 0.0833 | 0.5167 | 0.5167 | 0.45 | 0.0833 | 0.0167 |
| **P value summary** | ns | ns | ns | ns | ns | ns | * |
| **Exact or approximate P value?** | Exact | Exact | Exact | Exact | Exact | Exact | Exact |
| **Significant? (α = 0.05)** | No | No | No | No | No | No | Yes |


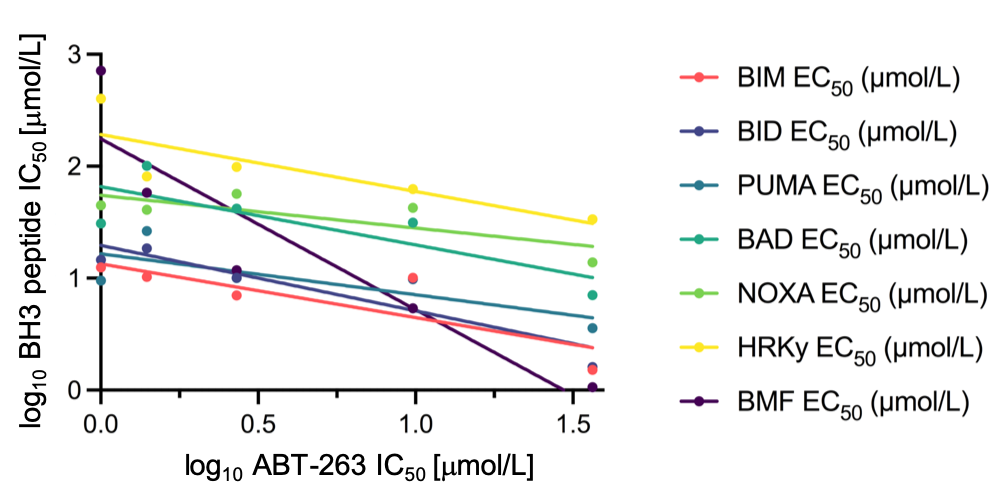


**Table S2. Correlation between mitochondrial priming (EC_50_ values for BH3 peptides-induced MOMP) and senolytic sensitivity (A1331852 IC_50_) in proliferative (untreated) and TIS A549 lung cancer cells.**

|  | **BIM** | **BID** | **PUMA** | **BAD** | **NOXA** | **HRK** | **BMF** |
| --- | --- | --- | --- | --- | --- | --- | --- |
| **Spearman r** | 0.9 | 0.9 | 0.4 | 0.4 | 0.5 | 0.9 | 1.0 |
| **P value** | 0.0833 | 0.0833 | 0.5167 | 0.5167 | 0.45 | 0.0833 | 0.0167 |
| **P value summary** | ns | ns | ns | ns | ns | ns | * |
| **Exact or approximate P value?** | Exact | Exact | Exact | Exact | Exact | Exact | Exact |
| **Significant? (α = 0.05)** | No | No | No | No | No | No | Yes |

**
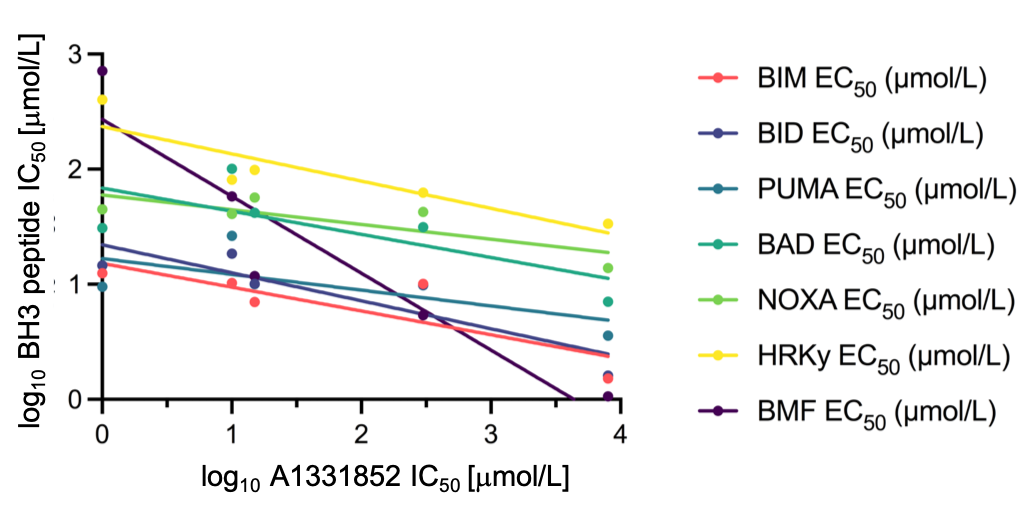
**

**Table S3. Correlation between mitochondrial priming (EC_50_ values for BH3 peptides-induced MOMP) and ABT-263/navitoclax senolytic indexes in proliferative (untreated) and TIS A549 lung cancer cells.**

|  | **BIM** | **BID** | **PUMA** | **BAD** | **NOXA** | **HRK** | **BMF** |
| --- | --- | --- | --- | --- | --- | --- | --- |
| **Spearman r** | -0,880 | -0,794 | -0,618 | -0,559 | -0,738 | -0,679 | -1,000 |
| **P value** | 0.049 | 0.109 | 0.266 | 0.328 | 0.155 | 0.208 | 0.017 |
| **P value summary** | * | ns | ns | ns | ns | ns | * |
| **Significant? (alpha = 0.05)** | Yes | Yes | No | No | No | No | Yes |


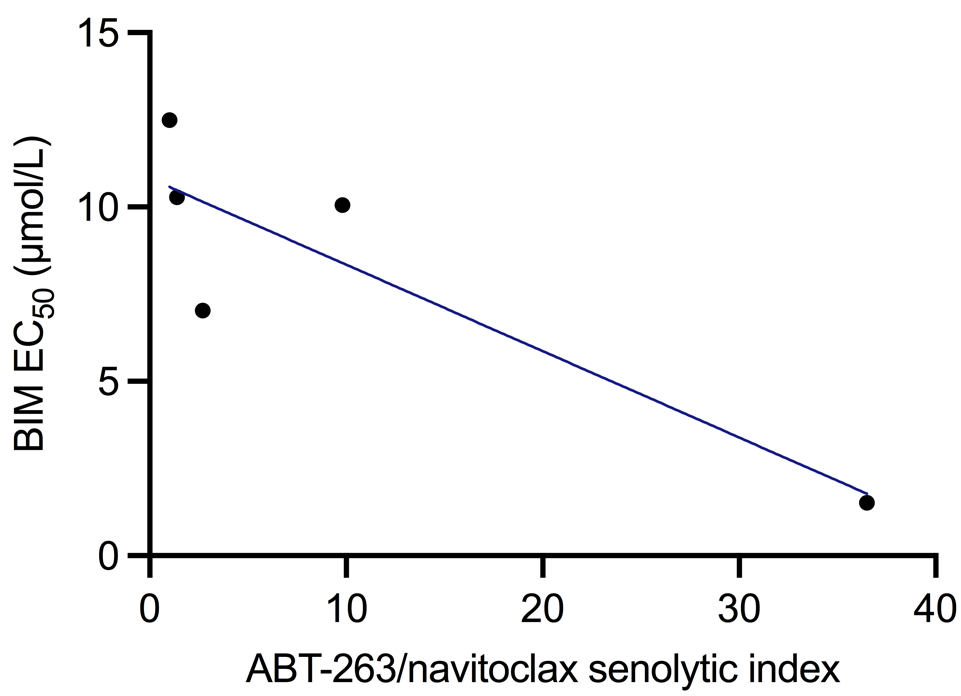


**Table S4. Correlation between mitochondrial priming (EC_50_ values for BH3 peptides-induced MOMP) and A1331852 senolytic indexes in proliferative (untreated) and TIS A549 lung cancer cells.**

|  | **BIM** | **BID** | **PUMA** | **BAD** | **NOXA** | **HRK** | **BMF** |
| --- | --- | --- | --- | --- | --- | --- | --- |
| **Spearman r** | -0,889 | -0,744 | -0,566 | -0,518 | -0,737 | -0,592 | -1,000 |
| **P value** | 0.044 | 0.149 | 0.320 | 0.371 | 0.155 | 0.293 | 0.017 |
| **P value summary** | * | ns | ns | ns | ns | ns | * |
| **Significant? (alpha = 0.05)** | Yes | No | No | No | No | No | Yes |


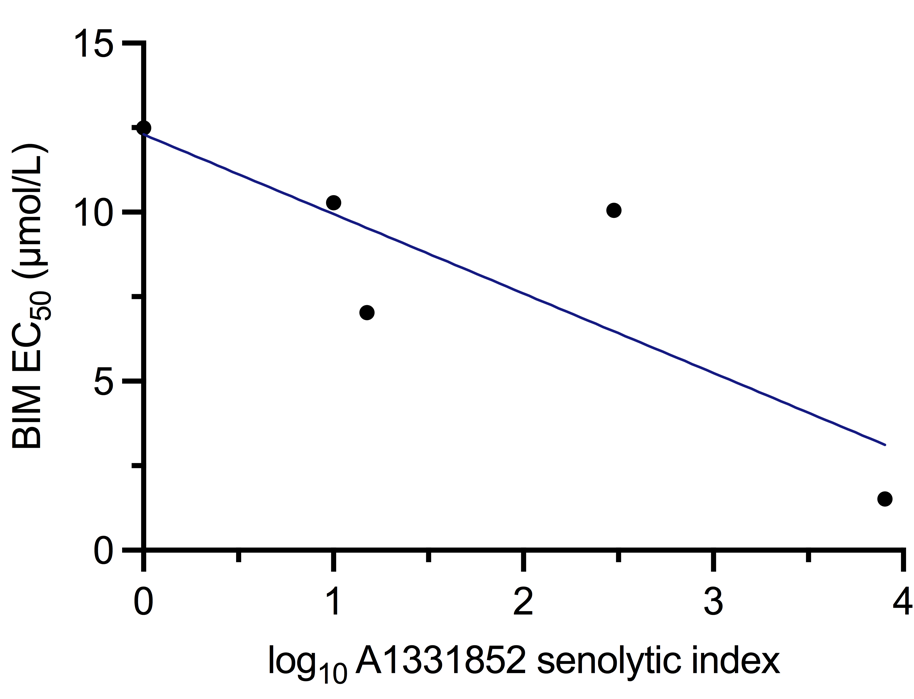


**2. SUPPLEMENTARY FIGURES**

**
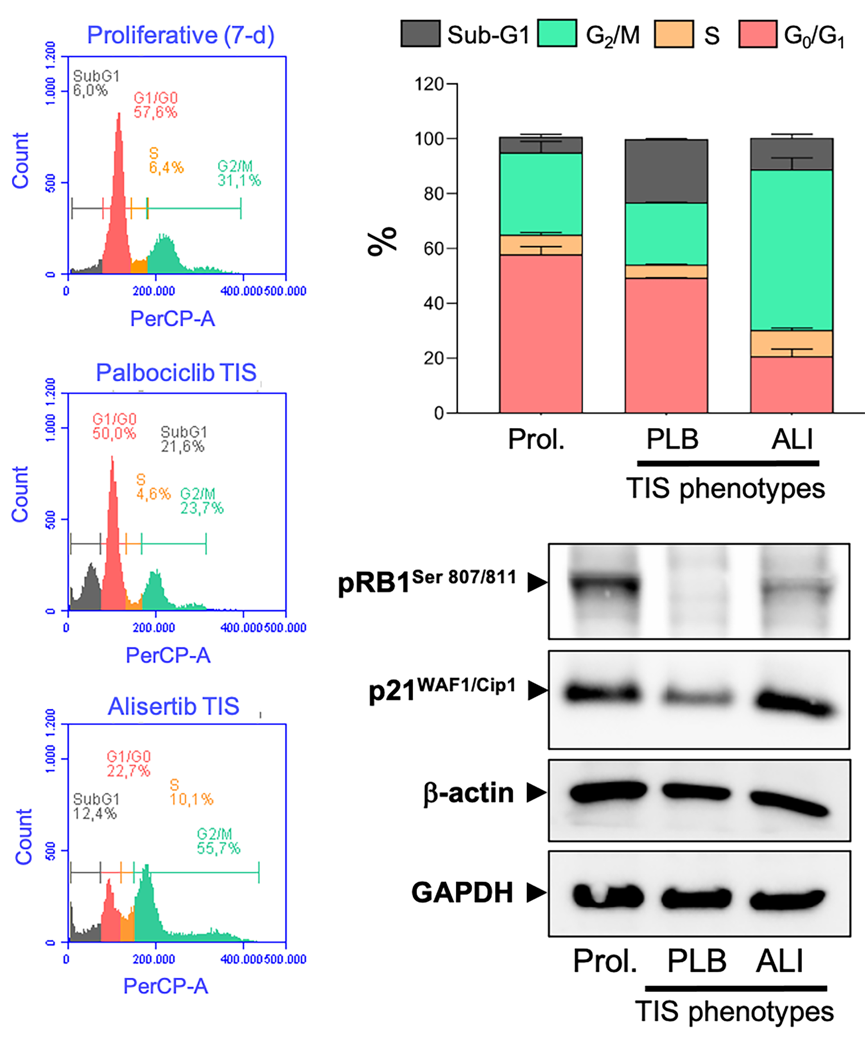
**

**Figure S1. Generation of TIS phenotypes in LoVo colon cancer cells.** Representative flow cytometry plots showing the gating of the cell cycle distribution of proliferative (untreated) and TIS phenotypes. The histogram shows the percentage (mean ± S.D., n=3) of cells in the four cell cycle phases as a function of the treatment condition. Expression levels of phospho-RB^Ser807/Ser811^ and p21^WAF1/Cip1^ were detected by immunoblotting in whole cell lysates of proliferative (untreated) and TIS LoVo cancer cells (7-d) using specific antibodies and β-actin/GAPDH as loading controls. The figure shows a representative immunoblot from multiple (n=3) independent experiments. PLB: Palbociclib; ALI: Alistertib.
