# Supplementary material for: Mitochondrial priming and response to BH3 mimetics in “one-two punch” senogenic-senolytic strategies
Source: Cell Death Discov. 2025 Mar 7;11:91. doi: 10.1038/s41420-025-02379-y (PMC11889205; doi:10.1038/s41420-025-02379-y)

Mitochondrial priming and response to BH3 mimetics in “one-two punch” senogenic-senolytic strategies

Uncropped original WBs

Figure 1A
A549

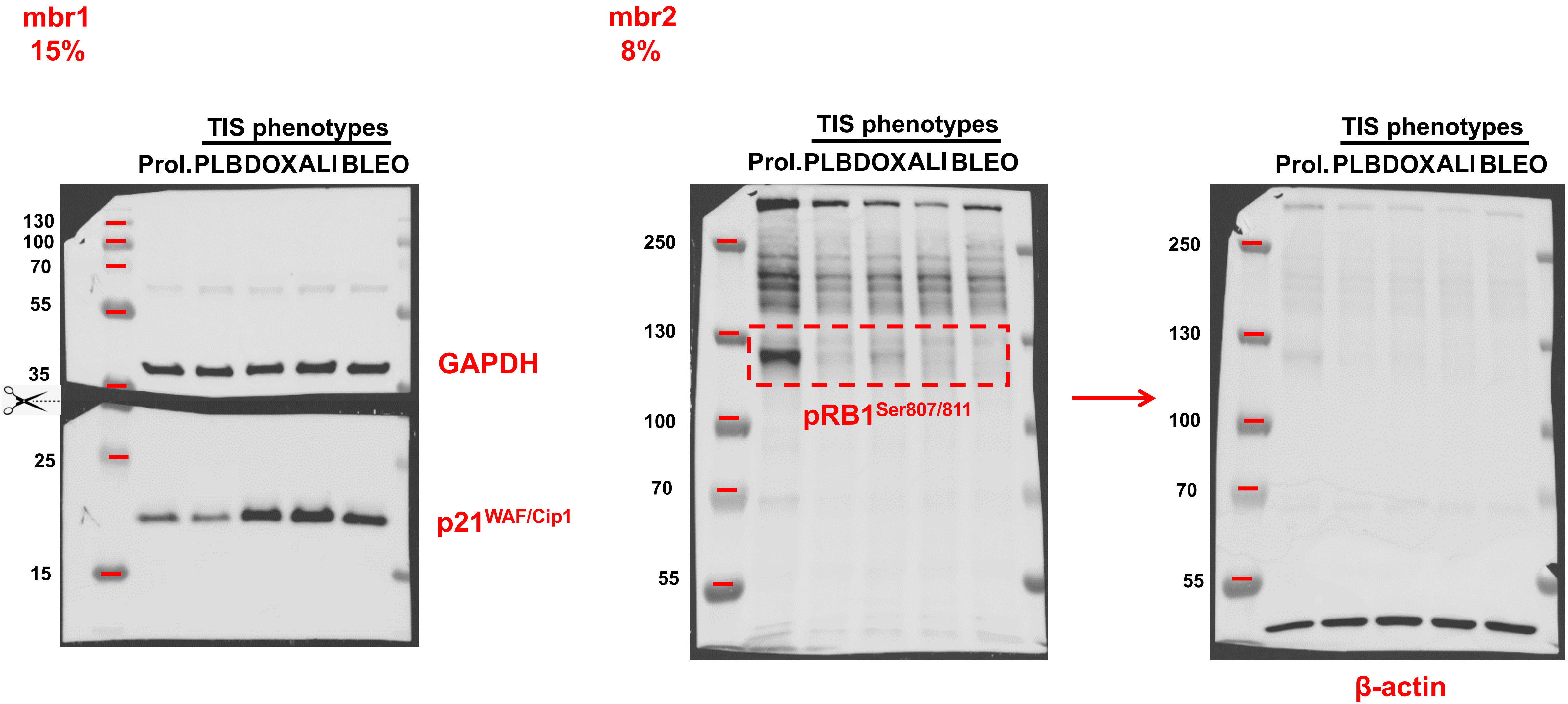

Figure 5A
IMEC

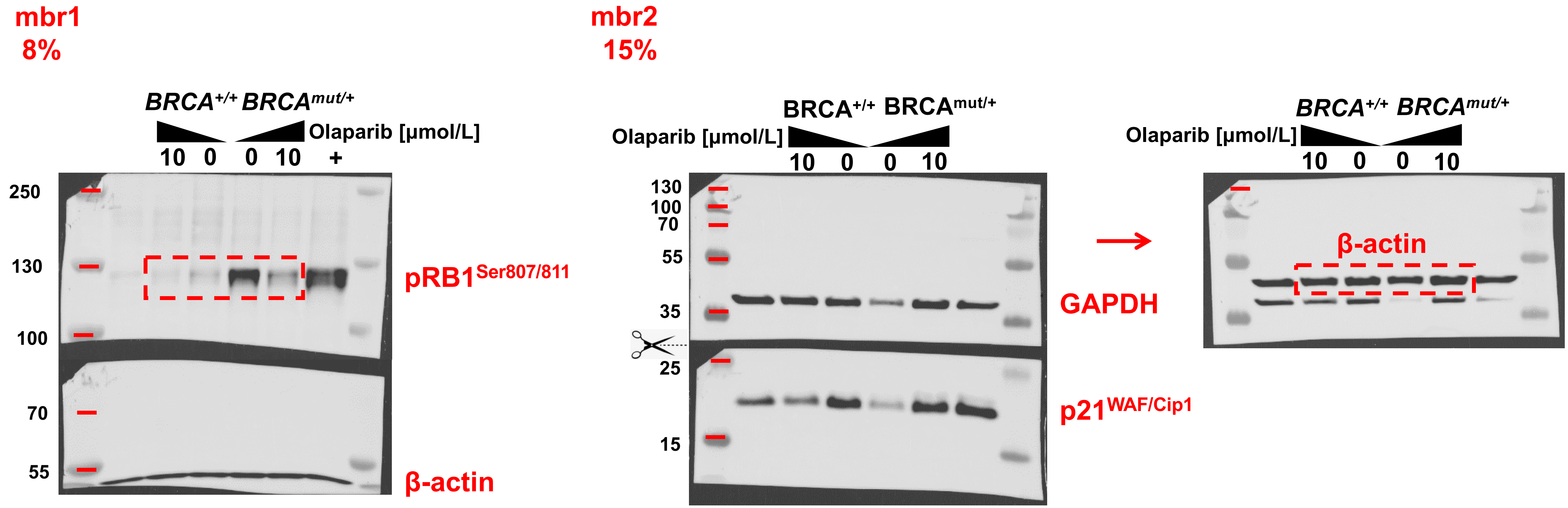

Figure S1
LoVo

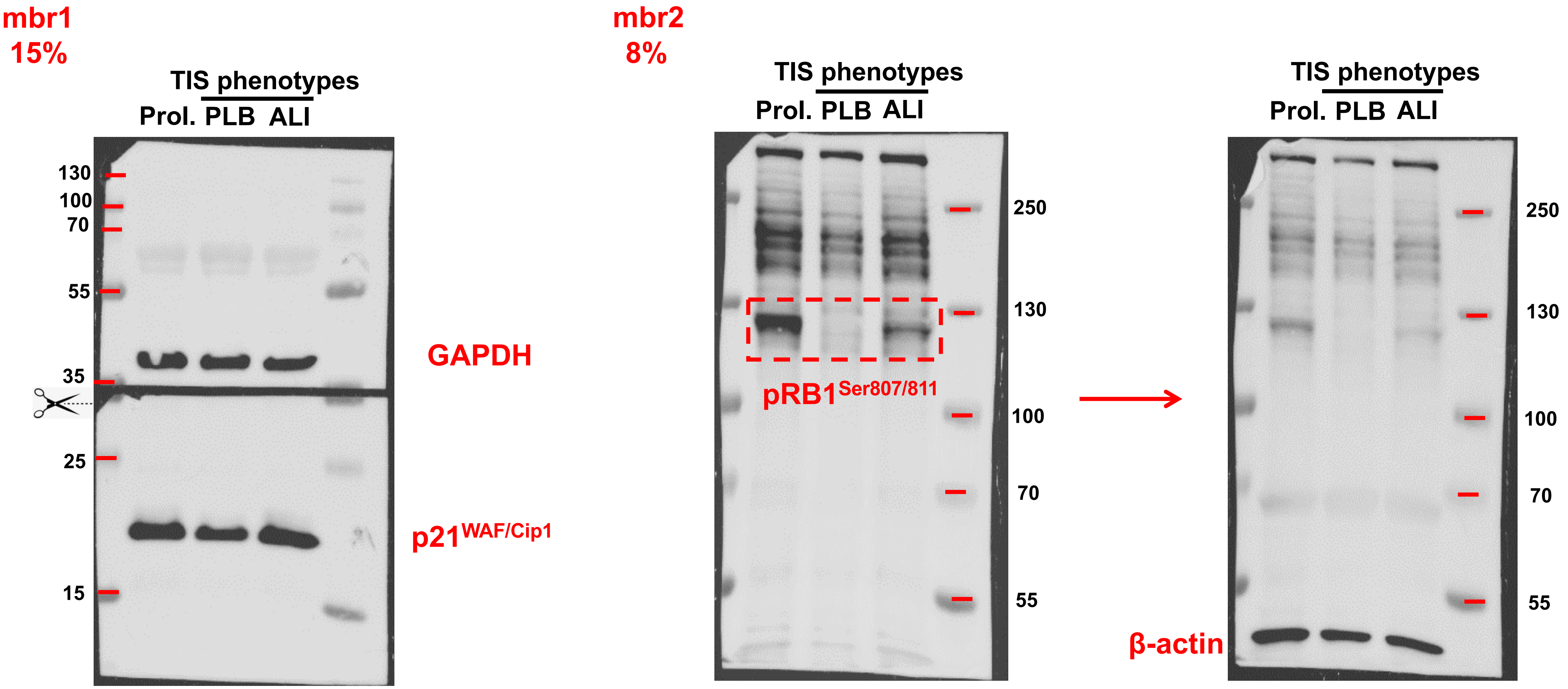

Supplement: Supplementary file 2 — Original data [file 41420_2025_2379_MOESM2_ESM.pdf]
